# Using GIS, remote-sensing and linear modeling to prioritize spatially explicit action against erosion and sediment transport within Lake Victoria watershed in Kenya

**DOI:** 10.1371/journal.pone.0359542

**Published:** 2026-10-01

**Authors:** Nickson Erick Otieno

**Affiliations:** National Museums of Kenya, Zoology Department, Nairobi, Kenya; Shiv Nadar University - Campus Delhi NCR: Shiv Nadar University, INDIA

## Abstract

Watershed degradation from human encroachment is a worldwide phenomenon and a keystone driver of erosion, transport and deposition of sediment material into downstream lakes and other water bodies. Despite global recognition of the growing scale of these linkages, comprehensive evaluation and ranking of the range of specific parameters associated catchment destruction and subsequent sediment movement, or those contributing to its mitigation, has rarely been undertaken. This study applied GIS with remote-sensing with linear modeling to assess influence of geomorphic and hydrological as well as land-use land-cover (LULC) elements in driving net erosion indicator (*NEI*), sediment transport indices (*STI),* and an index of overall erosion potential, *ErPot* combining *NEI* and *STI*. The LULCs were: crop cover, forest cover, wetland cover, urban-features cover while the geomorphic-hydrological parameters were slope gradient, elevation and sub-watershed size. This was undertaken across 10 delineated sub-watersheds forming the catchment of Lake Victoria on the Kenyan territory side. NEI was predicted to be enhanced by cop cover, urban development and slope angle; STI was enhanced by slope angle, sub-watershed upslope size. While ErPot increased with sub-watershed upslope size, NEI, STI and ErPot were all predicted to be mitigated by forest and wetland cover sizes. Using these results, the sub-watersheds of Busia-North, Rangwe and Homabay-North were considered priority for action to reduce net erosion indicator. Similarly, the Mara and Yala sub-catchments, and also the Mara the Yala and Nzoia sub-catchments were considered of greatest significance in prioritizing action to mitigate sediment transport and overall erosivity, respectively. These results, the first from modeling a trans-boundary freshwater catchment erosion, are practically applicable in decision making for designing a county-based strategy for habitat rehabilitation and conservation that specifically prioritizes the critical zones identified.

## Introduction

The growing global demand for food for the correspondingly escalating human populations continues to exert pressure on land-based agricultural systems in the backdrop of increasing challenges relating to dwindling soil qualities, high costs of production and changing weather patterns [1–4]. This has occasioned a trend in food production and supply chains towards increased exploitation of aquatic-based resources in to supplement those from agriculture so as to bridge gaps in demands at local, regional and global levels [5,6]. At the same time and a s a consequence, growing urban water scarcity is compelling more residents, city authorities and industries are relocating to areas closer to more permanent water sources [7–9]. In low-income economies, especially in tropical regions, this mainly takes the form of populations moving closer to lakes, dams, rivers and waterways [10,11]. Such encroachment, reclamation or conversion of riparian, catchments or watershed habitat often leads to increased risks of domestic, agricultural and industrial pollution but also siltation of waterbodies besides excessive water abstraction [12,13] as well as overexploitation of fisheries and other aquatic resources [14,15].

In particular, farming activity on riparian land or catchment zones is recognized as major source of agrochemical runoffs into surface and underground water reservoirs, and also transport and deposition of silt and other sediments and solid waste through streams or rivers [16,17]. Urban development and human settlement on these areas is similarly associated with solid and liquid effluent pollutants from domestic, industrial, construction and demolition activities [18]. The impacts of these activities tend to be significantly enhanced with increased slope gradient [19] and proximity to the water body itself such that pollutants and sediments have reduced likelihood for absorption, dilution or other forms of buffering before entering downstream water bodies [20, 21]. Such impacts are further exacerbated by other sporadic events and phenomena associated with impacts of climate change, such as flush floods and significant fluctuations in lake levels [4,22]. Additional potential consequences of watershed encroachment include introduction and influx of invasive species, overall declines in water quality and poor health of organisms [5,7,23,24], all of which have negative ramifications for aquatic food webs in general [25,26].

Although the related processes of surface erosion, sediment transport and deposition downstream is primarily a natural process [27–29] necessary for supporting general nutrient cycling and distribution [30], their physiographic, hydrological and ecological impacts are often profoundly exacerbated by a wide range of encroachment-related anthropogenic activities and land-use land-cover (LULC) forms. These effects may be more immediate and shorter-term from routine human activities along the shoreline and riparia, but may be significantly more profound and longer-term from activities at wider, more intensive scales in the catchment zones further upstream [31].

On a global scale, sediment transportation and general surface erosion is mainly attributed to vegetation removal and agricultural activities, typically characterized by frequent vegetation clearing and soil loosening. Industrial and urban development are also major triggers, especially in highly developed regions. Among its negative impacts are increased risks of coastal flooding, poor quality of drinking water, reduced health of aquatic species, diminished fisheries and reduced efficiency in maritime transport activities [27,32]. Regardless of the specific source-route, sediment erosion and movement rate or volumes can be significantly accentuated by increased slope gradient especially at higher altitudes [19,33,34]. For instance, a simulated study by Hatono et al.[35] estimated the global sediment transport phenomenon from catchments to be in the range of 3.0 to 4.6 billion tons annually. Nonetheless, such sediment removal and transport may also be considerably cushioned by rougher terrain, or buffered by enhanced vegetation cover or wetland habitats which serve to either reduce sediment size, per capita amount, or movement speed [33,36,37]. This is more likely so if such buffering features constitute a proportion of the drainage basin, a scenario which may at times mitigate the conventional expectation of direct proportionality between watershed area size and discharge volume of water as a medium of surface erosion.

Lake Victoria, the second largest freshwater lake in the world and the largest in the tropics [38], supports an estimated human population of 42 million who depend on it as a primary livelihood source in terms of food, water, employment and other goods or services [39,40]. Fishing activities constitute the main food source but also a key source of revenue for exchequers of the three East African states – Kenya, Uganda and Tanzania – but the rapidly growing dependency on the lake’s fisheries resources has resulted in considerable overfishing in recent years [41,42]. At the same time, the lake continues to be impacted by other human activities in and around it, including pollution, invasive fauna and flora, and rapid riparian development activities [43]. Further upstream, the catchment continues to be degraded through deforestation, agricultural expansion, human encroachment, urbanization and excessive water abstraction, resulting in increased silt loading, solid waste pollution and eutrophication from agricultural, domestic and industrial chemical runoff [12,13]. The consequent degradation of the lake, compounded with impacts of climate change, is associated with now more readily noticeable phenomena of lake level fluctuations, low water quality and poor health of fisheries which is demonstrated by, declining trends in fish landing volumes [44,45].

The novelty of this paper is the trans-boundary nature of the habitat studied, the simultaneous considerable or multiple parameters influencing both catchment erosion and sediment transport processes; and the evaluation of the relative role of positive drivers vs. mitigatory parameters, especially with incorporation of predictive modeling. The specific novelty is in simultaneously incorporating multiple LULC and geomorphic-hydrological parameters in the erosion equation with the deliberate aim of evaluating the relative effects of the positive drivers vs. the mitigating factors, on the erosion parameter itself, then using statistical modeling to compare across sub-watersheds to guide spatial prioritization for mitigation action. Most previous studies examined just single parameters in isolation, and even then just in one response direction.. Only a few studies have evaluated the roles of more than one LULC parameter that might potentially enhance or mitigate STI [19–21,37]. Therefore, this study is novel because it is the first one to assess STI using a comprehensive range of more than 5 LULC parameters, while concurrently also incorporating the context of modeling for scenario prediction (rather than spot-mapping) of its contribution to overall potential for surface erosion in combination with net surface erosion. It is also unique in being the first to apply this approach for a cross-border lentic habitat in general and Lake Victoria in particular. Furthermore, this study area forms part of the wider African Great Lakes and Eastern Rift Valley system considered by UNESCO as a regional hotspot for erosion and sediment transportation, making the results more significant for potential adoption or practical application in a national or regional environmental, hydrological or strategic-planning perspective.

The overall aim of this study was to apply geospatial analysis and linear modeling to evaluate relative influence of a range of topographic and LULC factors that either promote or mitigate the scale of surface erosion and sediment movement across 6 major sub-watersheds of Lake Victoria on the Kenyan territorial side, for the purpose of identifying priority zones in which to focus potential efforts for actions that promote sustainable management of the lake’s watershed. The specific objectives were:

1. Establish a classification of Lake Victoria catchment area based on sub-watershed-level ranking of surface erosion, sediment transport indices, and a combination of both;
2. Create predictive linear models for key geomorphic/topographic and LULC parameters driving net surface erosivity, sediment transportation, and a combination of both
3. Highlight the most important geomorphic and LULC parameters that either drive or mitigate overall erosivity potential to guide action-based prioritization for sustainable catchment management effort

We generally expected to find that, when all geomorphic and LULC parameters are kept constant:

a) Sub-watershed area size would be directly proportional to each of the three erosion and sediment transport indicators (NEI, STI and ErPot)
b) The sub-watersheds with the largest upslopes would contribute more to the scale of overall erosion and sediment transportation, owing primarily to their correspondingly larger contributing surfaces;
c) Erosion and sediment transport processes are less profoundly influenced by land-use-land-cover (LULC) than by topographic and geomorphic parameters, given that the latter are relatively less dynamic and longer-term in nature

## Methodology

### Study area

Lake Victoria is the second largest freshwater lake in the world, straddling the 3 east African states of Kenya, Uganda and Tanzania (Fig 1). It covers a total area of 68.800 Km^2^, of which 6%, 45% and 49% lie in Kenya, Uganda and Tanzania, respectively [46] with depth ranging from 0−80 m, and averaging 40 m [47]. On the Kenyan side, the lake’s riparian and catchment zones are dotted with several urban areas of varying sizes and human populations, the main ones being the city of Kisumu and the towns of Homa Bay, Siaya, Busia and Migori (Fig 1) [48]. The study focused on the region bounded by 19 counties of the western Kenya region covering 01°33´S-01°7´N and 33°58´E-38°10´E, an area of some 51,200 km^2^ [49] and which constitute the main lake’s watershed on the Kenyan territorial side (Fig 1). This is a large and diverse area both topographically and climatically, with mean elevations ranging from 1,900−13,400 m above sea level, a mean annual rainfalls between 900–2,200 mm, mean temperatures between 14° C around Mt Elgon region in Bungoma County to 32° C in parts of Keiyo-Marakwet County [50]. Therefore the constituent counties do not necessarily share much environmental uniformity in any broad sense. Several of the extensively hilly, mountainous and other highly-elevated areas within this region constitute the western-Kenya zone of the country’s most strategically important natural water sources, also known as Water Towers (KNBS, 2023).

**Fig 1:**
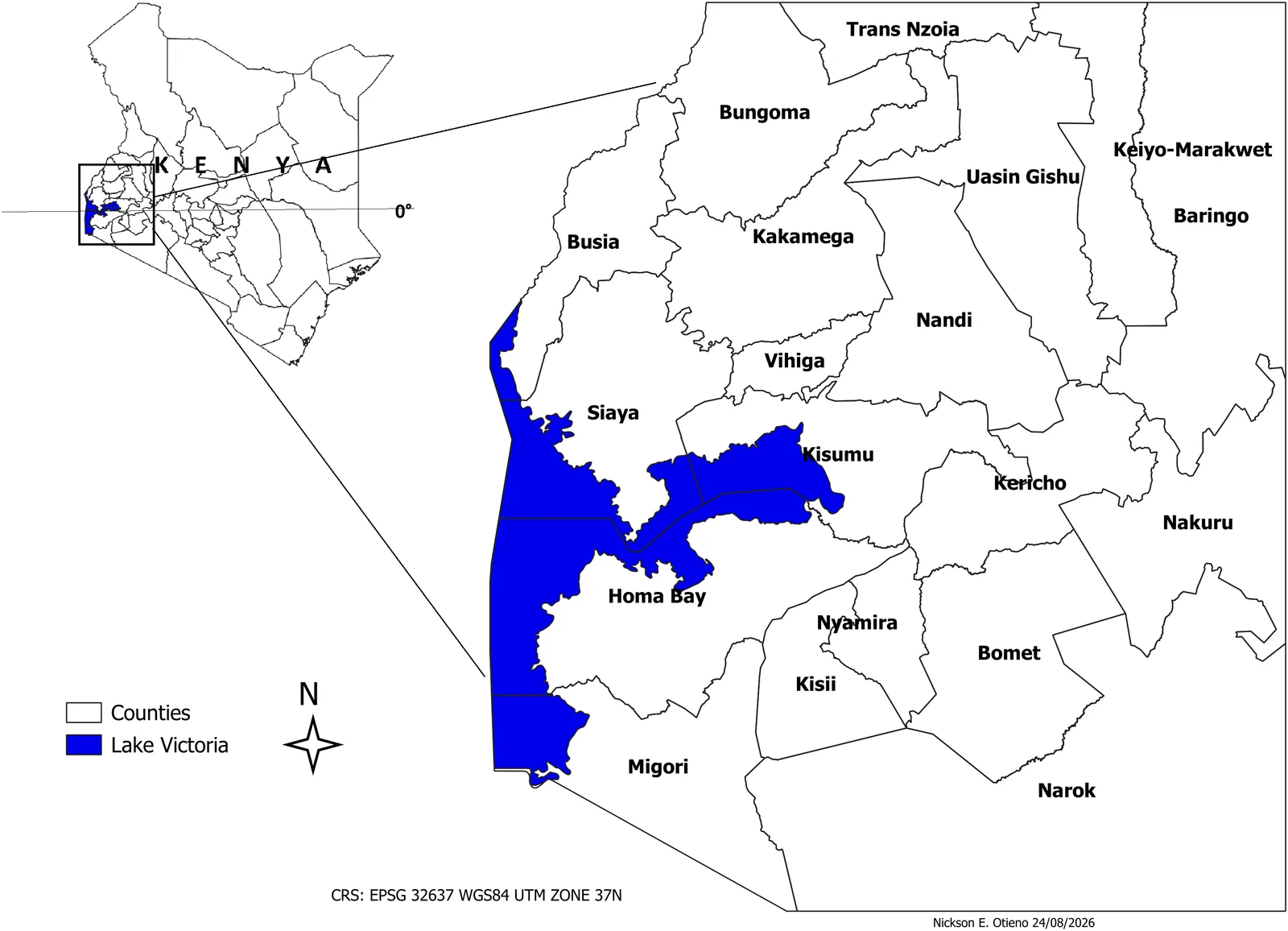
Map of study area indicating the focal area in Kenyan side of Lake Victoria encompassing the counties which form the catchment of the lake (Courtesy: Google Maps, 2026).

### Spatial data acquisition and processing

Spatial data were obtained from diverse sources including the Kenya Meteorological Department, the World Resources Institute Open Data (majority of the shapefiles); the Kenya National Bureaus of Statistics (KNBS); the East African Regional Center for Mapping Of Resources for Development; RCMRD (the Kenya crop-cover mask layer); the County Government of Kisumu; Friends of Lake Victoria (OSIENALA); and Google Maps archives. All data were processed on a quantum geographical information systems (QGIS v. 3.44.6) platform. Satellite imagery data were acquired as Landsat 8 Level 1 imagery of land cover data from *Earth Explorer* [51], comprising a total of 4 scenes of 90 m resolution images for 2025. Multiple panels of the (or acquired scenes were composited into one mosaic raster layer matching the extent of the selected focal study region of the Lake Victoria watershed (Fig 2 A 1–3). From a 90-m spatial resolution srtm digital elevation model [DEM] of Kenya reprojected to the region’s UTM zone value, a clipping was conducted, masking with a vector layer of Kenya counties, labeled accordingly for each County. Using the QGIS’s SAGA 9.2 add-on processing tool, the hydrological and terrain analysis functions were applied in filling sinks in the clipped DEM, in establishing Strahler-ordered steam channel networks, with the minimum threshold value capped at 6 (Fig 2A-3, 2B), and in identifying major confluents of rivers at the points where they enter into Lake Victoria [Fig 2A-3]. The clipped layers were subsequently re-projected from Lat/Long coordinate reference system to UTM zone 37N for Kenya (Fig 2B).

**Fig 2:**
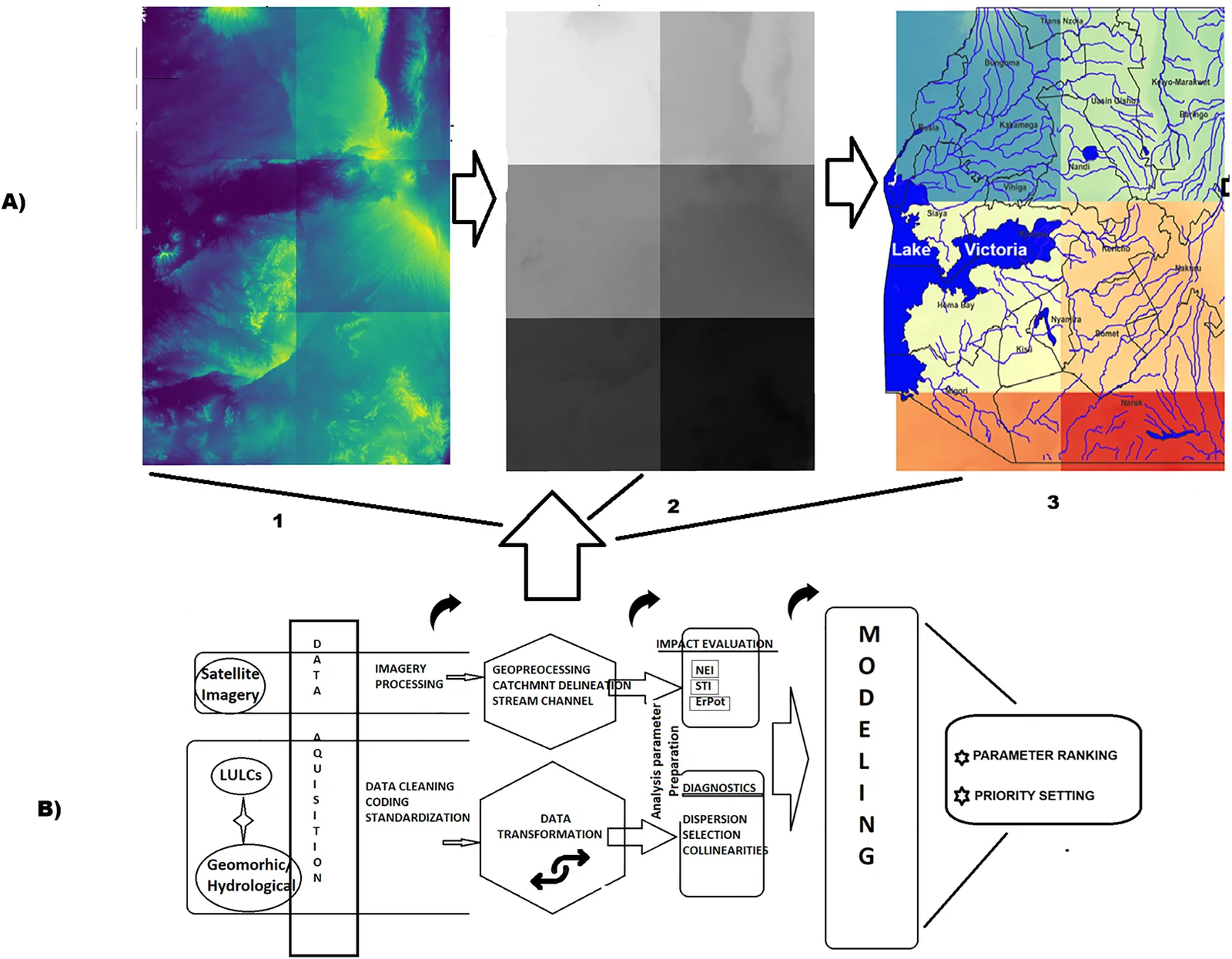
A) The geo-processing stages in development of the mosaiced panels of the satellite imagery scenes in 1-2 pseudo-colors and 3) Indicating the Kenyan counties intersected along with the associated stream channel network leading to Lake Victoria; B) Schematic flow diagram summarizing the analytical framework adopted in the study.

**Fig 3:**
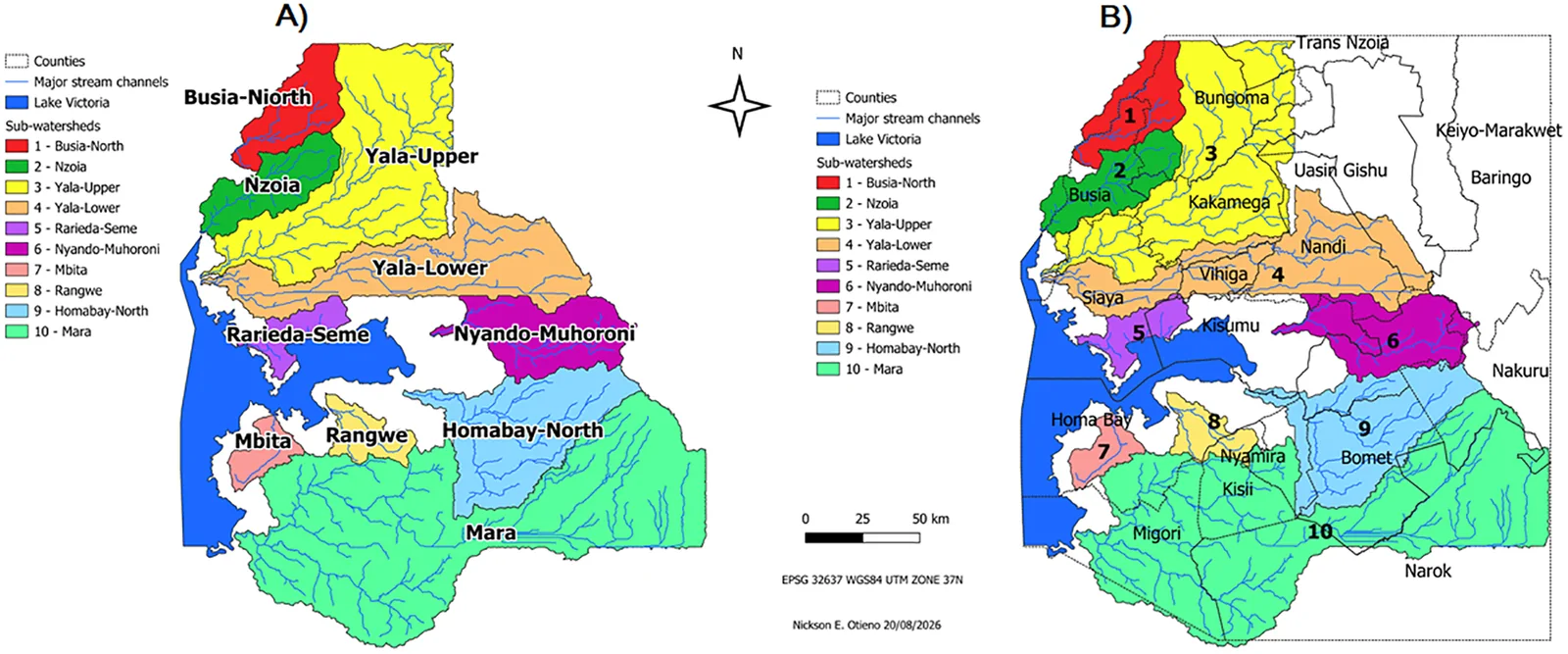
Representation of locations and sizes of the then sub-watershed zone) and their respective channel networks A) Separately and B) Indicating the Kenyan counties with which they intersect and overlap.

The combination of stream channels and major rivers were therefore used for delineating the main sub-watersheds, and in calculating the respective upslope area sizes. The individually delineated catchment zones and upslope area sizes were then vectorized and combined using the merge procedure, into one vector layer. The merged upslopes were dissolved to consolidate the total upslope areas into one for the entire watershed, before using join by attribute (summation) and zonal statistics to derive total upslope sizes delineated for the distinct sub-watersheds, using the Kenya counties map as an overlay vector. A similar process of clipping and masking was employed in processing an additional array of complimentary datasets with the respectively corresponding additional attributes for each of the counties. These additional attributes included information on area-size cover of natural and plantation forest; cropland; urban development features and infrastructure including road networks; and wetland and swamp habitat. The second set of raster datasets of the clipped and re-projected 90-m spatial resolution srtm DEM and hillshade, was used to derive data on slope gradient, terrain ruggedness indices (TRI) and general elevation for each sub-watershed [52,53].

### Statistical analyses

#### Net erosion indicator (NEI).

The fundamental physical and ecological role of each of the key selected topographical and LULC parameters was taken into consideration in calculating the rank value of each sub-watershed for its role in contributing to overall water-mediated erosion towards Lake Victoria. This role rank was determined as the net erosion indicator (NEI) which is fundamentally attributable to removal of surface soil and other suspended sediment material by water through the network of stream channels and associated rivers. In this study, it was established as the ratio of the product of key parameters that primarily promote surface erosion, to that of the product of those parameters that potentially serve to mitigate erosion through retention of eroded material [19, 37,54,55]. This function was expressed in the relationship:


$$\begin{array}{c} NEI\;=\frac{\left[(TUA*Slope*Elevation*Cropsize*Urban)\right]}{\left[(TRI*Forest*Wetland)\right]} \end{array}$$

*Or*


$$\begin{array}{c} NEI\;=\;[logTUA+logSlope+logElev+logCrop+logUrban]-[logTRI+logForest+logWetland] \end{array}$$

Where *NEI* = net erosion indicator; *TUA* = total area size of the sub-watersheds delineated; *Slope* = Mean slope gradient; *Elev* = mean elevation; *Crop* = total size of area covered by crop-based agriculture; *Urban* = total area covered by urban development and other infrastructure; *TRI* = terrain ruggedness index (elevation difference between adjacent pixels as outlined by Riley et al 1999^FFF^); *Forest* = total size of area covered by perennial woody plants; *Wetland* = percent area covered by wetland habitat. Prior to executing the formula, data were standardized through re-scaling by use of logarithmic transformation [56]. The selection of this range of topographic and LULC parameters for determining net erosion indicator was guided by the range of variables outlined by the United Nations Educational, Scientific and Cultural Organization (UNESCO) International Sediment Initiative’s assessment report on key drivers of erosion and sediment movement across regions of the world that are regarded as the global erosion hotspots [33].

### Sediment transport index (STI)

For each sub-watershed, the sediment transport index (the downward movement of soil particles and other material of various sizes [36,57], with or without agency of water – including other geomorphic processes that loosen the earth surface and trigger downward movement of suspended and non-suspended material under gravity – such as landslides, tremors, cultivation, construction or demolition activity) was calculated from the relationship:


STI = [A / 22.13]^0.6 * [sin[β] / 0.0896]^1.3

where ‘*A*’ = the contributing area within the delineated stream channel upslope area (i.e., size of the region contributing to the upslope per unit contour length); and *‘****β****’* = slope angle or gradient in degrees [58,59]. STI values were determined separately for each sub-watershed area, clipped off from the flow direction raster of the entire watershed. The flow accumulation raster was itself derived the watershed slope raster, corrected to remove zero or *nodata* values (QGIS Development Team (2026).

### Determining overall erosivity potential (ErPot)

The overall erosivity potential for each sub-watershed was calculated as the combined sum of the net erosion indicator (NEI]) and sediment transport index (STI). It represented removal and downstream movement of eroded material both by water and by other processes, and was determined from the relationship:


$$\begin{array}{c} ErPot\;=\;NEI\;+\;STI \end{array}$$

where *ErPot* = overall potential of the sub-watershed to contribute to erosion and transportation of eroded material towards Lake Victoria; *NEI* = the net erosion indicator; *STI* – sediment transport index The

### Modeling influence of topographic and LULC parameters on erosion and sediment transport

Predictive statistical models were developed for assessing linkage patterns between the topographic features and LULC parameters on one hand, and overall potential incidences or severity of net surface erosion (NEI) and sediment transport (STI) on the other, both independently as well as in combination. Here, generalized linear mixed models (GLMM) were used in *R v 4.4.2* programing language within the *lme4* and *multcomp* packages [60,61]. These were conducted, in successive turns, for NEI, *STI* and then overall erosivity potential (*ErPot = NEI + STI).* The error distribution family used was beta, with the *logit* link function. In each case, sub-watershed identity was included as a random factor, due to the observable considerable variations in this variable./ The other topographic and LULC elements were treated as fixed effects [61]. Since tests on spatial autocorrelation of the variables did not indicate any significance, sub-watershed identity was not included as an additional random factor [62].

Prior to analyses, variables were inspected for multi-collinearity, using the variance inflation factor (VIF) procedure through the function *vif* from the package *car* [63], setting a maximum VIH threshold of 4. No variables were found to be significantly collinear (S1 Fig). For each case, full models were first fitted with all variables retained after VIF, and then model fit diagnostics were conducted to test for data dispersion using the *glmmTMB* function within the *DHARMa* procedure in the *AER* package, and it was confirmed through plots of quantile-quantile and the rank-transformed DHARMa dispersion residuals (S2 Fig), that there were no significant overdispersions in each case [64–66]. Subsequently, a dredge process was applied within the *MuMln* package to select the best models stepwise based on Akaike Information Criterion (AIC) modified for small samples ((AICc), setting a threshold of ΔAIC ≤ 2 [67]. A total of 27 candidate models with multiple combinations were considered, (see S1 File for the best models selected with the respective retained predictor parameters). Model selection was followed by use of the function *model.avg* in package *AICcmodavg* to select the best conditional model subsets (package *glmulti)*, before evaluating conditional R squares for the remaining model subsets with the *r.squares* function, repeating this for each response variable at a time [67]. All significant effects were evaluated at p ≤ 0.05.

### Ethics approval and informed consent

As the study did not involve any handling of animal or human subjects, no formal ethical approval was required from the Ethics Committee of the National Museums of Kenya or the Kenya Wildlife Service. Informed consent was obtained verbally from relevant administrative representatives of all the respective counties included in this study.

## Results

### Stream channel network and sub-watershed) delineation

A total of ten main sub-watershed was delineated, constituting the key or upslope areas of various sizes associated with corresponding major rivers draining the Lake Victoria watershed (or catchment) on the Kenyan territorial side (Table 1, Fig 3).

**Table 1:** Sizes and other general attributes of the size major sub-watershed zones delineated and identified as potentially contributing to sediment transport into Lake Victoria.

Serial number	Sub-watershed name	Total upslope area size (km^2^)	Main associated river	Number of counties intersected
10	Mara	10919.37	Mara	7
3	Yala-Upper	5691.52	Yala	8
4	Yala-Lower	4051.67	Yala	8
9	Homabay North	3315.78	Awach/Sondu	5
7	Mbita	520.37	Awach/Sondu	1
8	Rangwe	661.96	Awach	2
5	Rarieda-Seme	765.80	Yala	1
6	Nyando-Muhoroni	2033.09	Nyando/Kusa	4
2	Nzoia	1368.97	Nzoia	3
1	Busia-North	1325.89	Nzoia	4

### Sub-watershed rankings for their contribution to overall NEI, STI and ErPot

The rank profiles for each of the sub-catchments in terms of their contribution to net erosion indicator (NEI), sediment transport index (STI) and overall erosivity potential (ErPot), are as shown in Fig 4, generally demonstrating that topographic and LULC parameters contribute in different ways and magnitudes to NEI, STI and ErPot. Sub-watershed are size appeared to be more important in determining sediment transport and overall erosivity potential than it impacted net erosivity index alone. Implicitly these ranks reflect corresponding sub-watershed-level rank priorities for potential action aimed at rehabilitating habitat for overall conserving the wider overall lake catchment.

**Fig 4:**
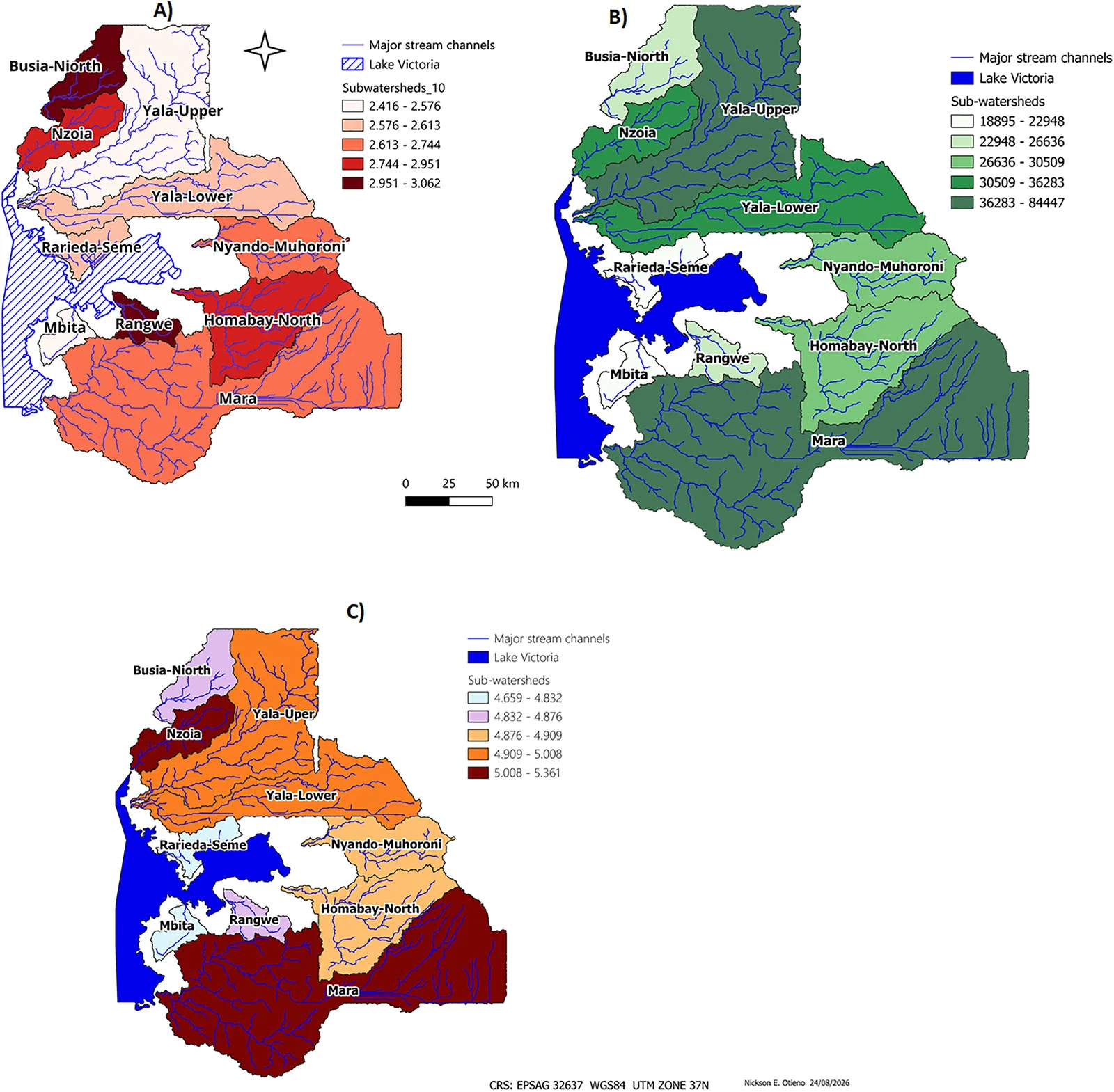
Sub-watershed rankings for A) Net erosion indicator (NEI); B) Sediment transport indices (STI); and C) Overall erosivity potential (ErPot).

### Predictive models of topographic and LULC parameter influences

#### Independent effects of NEI and STI.

Whereas slope angle, area covered by crops and urban infrastructure were predicted to enhance net erosion indicator index (Table 2, Fig 5 A-C), degree of terrain unevenness (terrain ruggedness index) size of forest cover and presence of wetland habitat were predicted to mitigate net erosion across all sub-watersheds (Table 2, Fig 5 C-F). Height above sea level did not appear show any significant predicted linkage to net erosion.

**Table 2:** Results of generalized linear mixed tests for predicted trends of net erosion indicator (NEI) and sediment transport index (STI) in response to the various scenarios of topographic and LULC parameters for the final selected models. *TRI* = Terrain ruggedness index; *Slop*e = General slope gradient; *Elevation* = Average height above sea level. Values in bold face are significant at p ≤ 0.05.

Response variable	Predictor variable	Parameter estimate	Standard error	Z statistic	*p*	Conditional R square
Net erosion indicator (NEI)	TRI	−0.055	0.001	−18.312	**0.035**	0.99.1
	Slope	0.945	0.325	2.907	**0.014**	
	Elevation	0.013	0.020	0.665	0.520	
	Forest cover	−0.045	0.009	−5.019	**0.002**	
	Crop cover	1.008	0.015	66,326	**<0.001**	
	Urban cover	0.022	0.001	16.746	**0.038**	
	Wetland cover	−0.054	0.005	−10.233	**<0.001**	
	Sub-watershed area	0.483	0.150	3.224	**0.048**	
Sediment transport index (STI)	TRI	−0.346	0.271	−1.279	0.291	0.97
	Slope	1.291	0.171	7.534	**<0.001**	
	Elevation	0.254	0.255	0.996	0.393	
	Forest cover	−0.391	0.065	−24.770	**<0.001**	
	Crop cover	−0.054	0.336	−0.161	0.883	
	Urban cover	0.197	0.128	1.536	0.222	
	Wetland cover	−0.087	0.027	−3.269	**0.005**	

**Fig 5:**
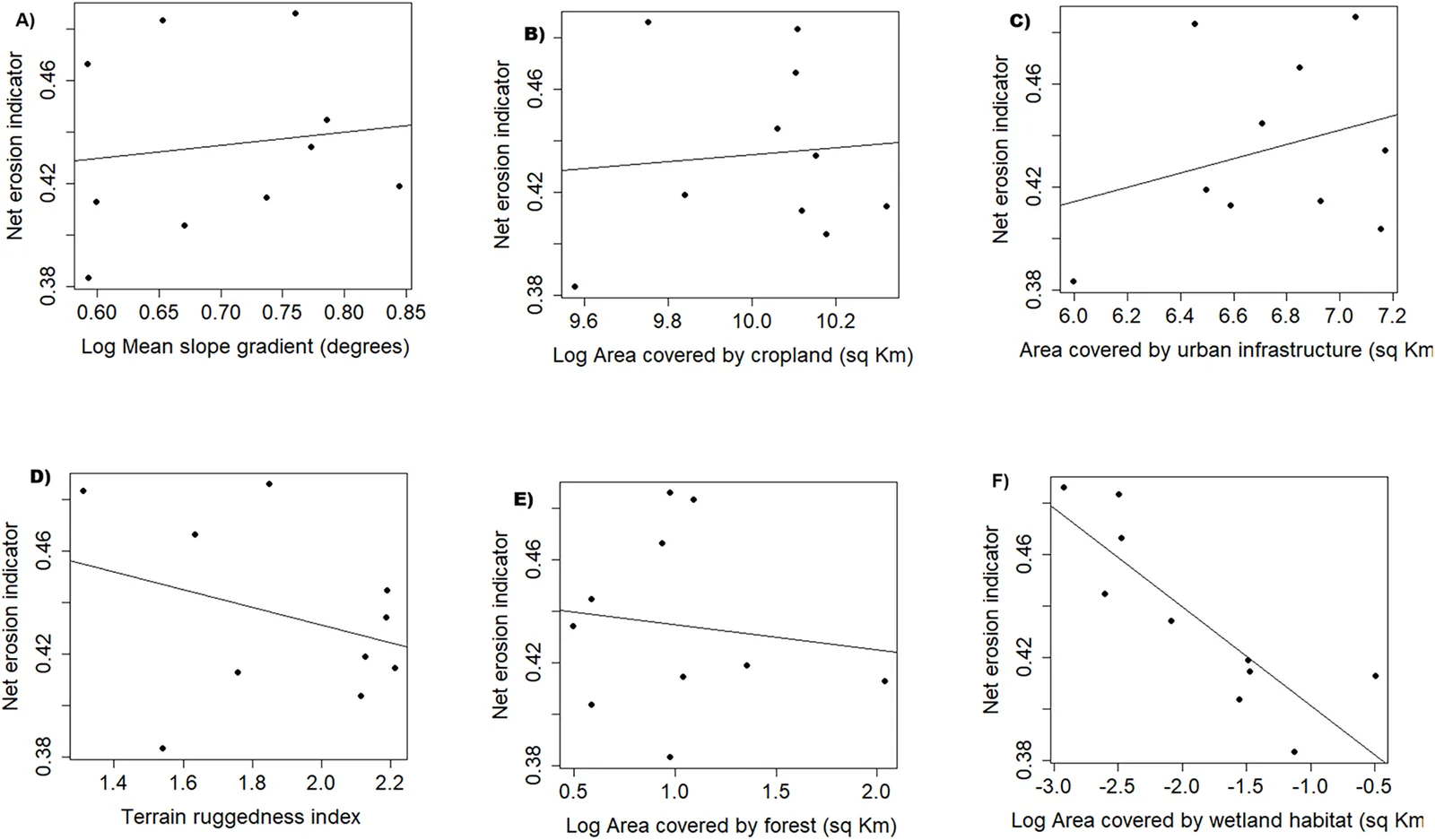
Regression plots of the predicted trends in net erosion indicator response to A) Slope gradient; B) Size of cropland; C) Size of urban infrastructure; D) Terrain ruggedness; E) Size of forest cover; and F) Size of area covered by wetland habitat.

On the other hand, sediment transportation was predicted to be enhanced by larger sizes of sub-watershed contributing material as well as slope angle (Table 2; Fig 6 A and B) while it was mitigated by extent of forest cover and area covered by wetland habitat (Table 2; Fig 6 C and D). The other variables did not show any predicted bearing on sediment transport scales.

**Fig 6:**
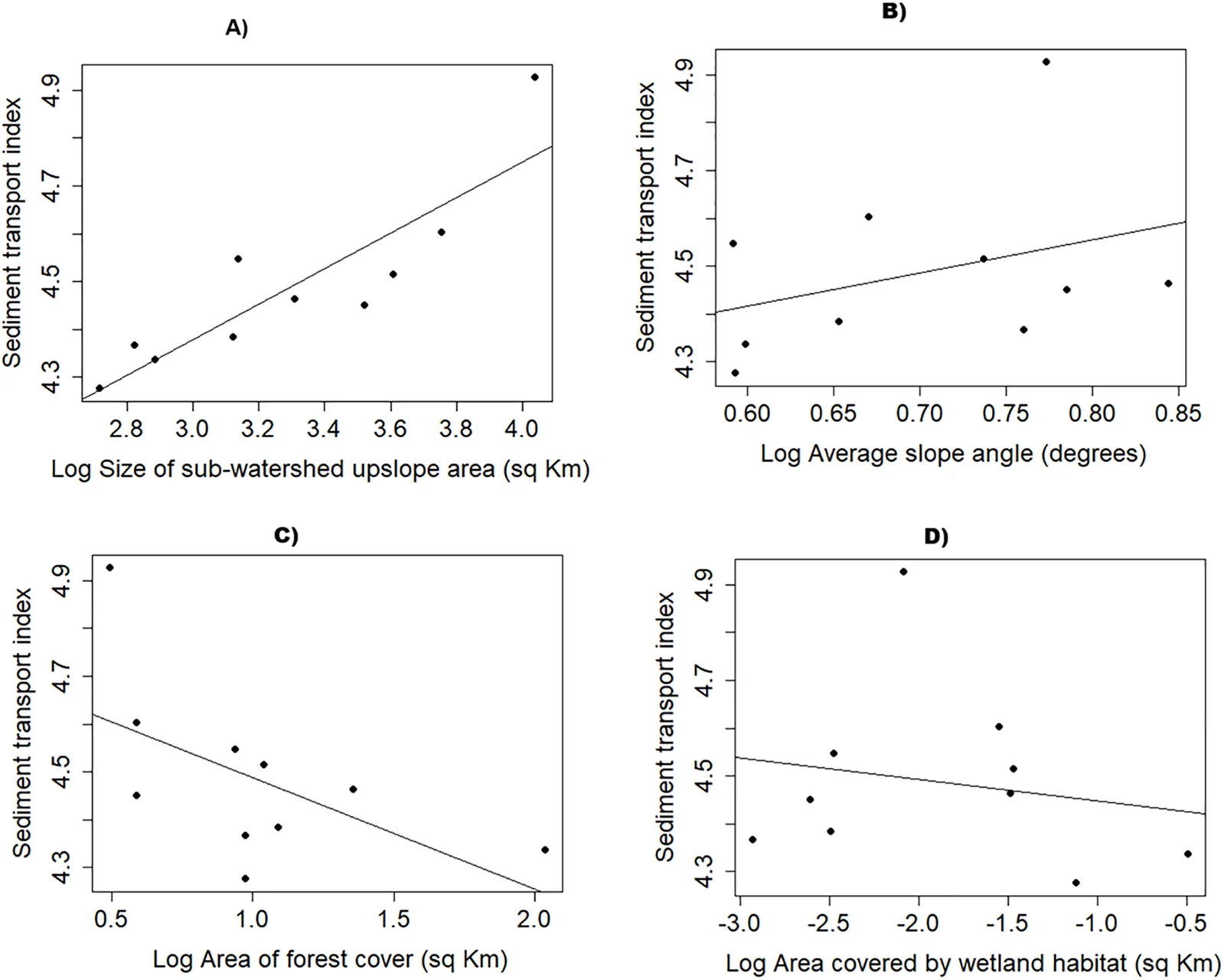
Regression plots of predicted trends in sediment transport index response to A) Sub-watershed area size[B) Slope size; C) Area covered by forest; and D) Total area covered by wetland habitat.

### The effects of erosion potential (ErPOT) – NEI and STI combined

The overall potential for erosion and sediment transport, representing the combination of NEI and STI showed a predicted positive response primarily to size of sub-watershed area and extent of crop cover but also height above sea level and urban development (Table 3; Fig 7A-D), but was predicted to be significantly mitigated only by total area covered by forest (Table 3; Fig 7E). Thus in general, crop cover was the most important cross-cutting driver parameter for erosion and sediment transport, whereas forest cover and wetland habitat were most important mitigating factors.

**Table 3:** Results of generalized linear mixed tests for predicted trends of overall erosivity potential (ErPot) in response to the various scenarios of the topographic and LULC parameter for the final selected models. *TRI* = Terrain ruggedness index; *Slop*e = General slope angle; *Elevation* = Mean elevation. Values in bold face are significant at p ≤ 0.05.

Predictor parameter	Parameter estimate	Standard error	Z statistic	p ≤ 0.05	Conditional R square
Sub-watershed	0.453	.150	3.015	**0.047**	
TRI	0.001	0.038	0.042	0.967	0.895
Elevation	0.945	0.325	2.907	**0.014**	
Forest cover	−0.243	0.120	−2.021	**0.047**	
Crop cover	1.008	0.015	66.326	<**0.001**	
Urban cover	0.007	0.003	2.113	**0.048**	

**Fig 7:**
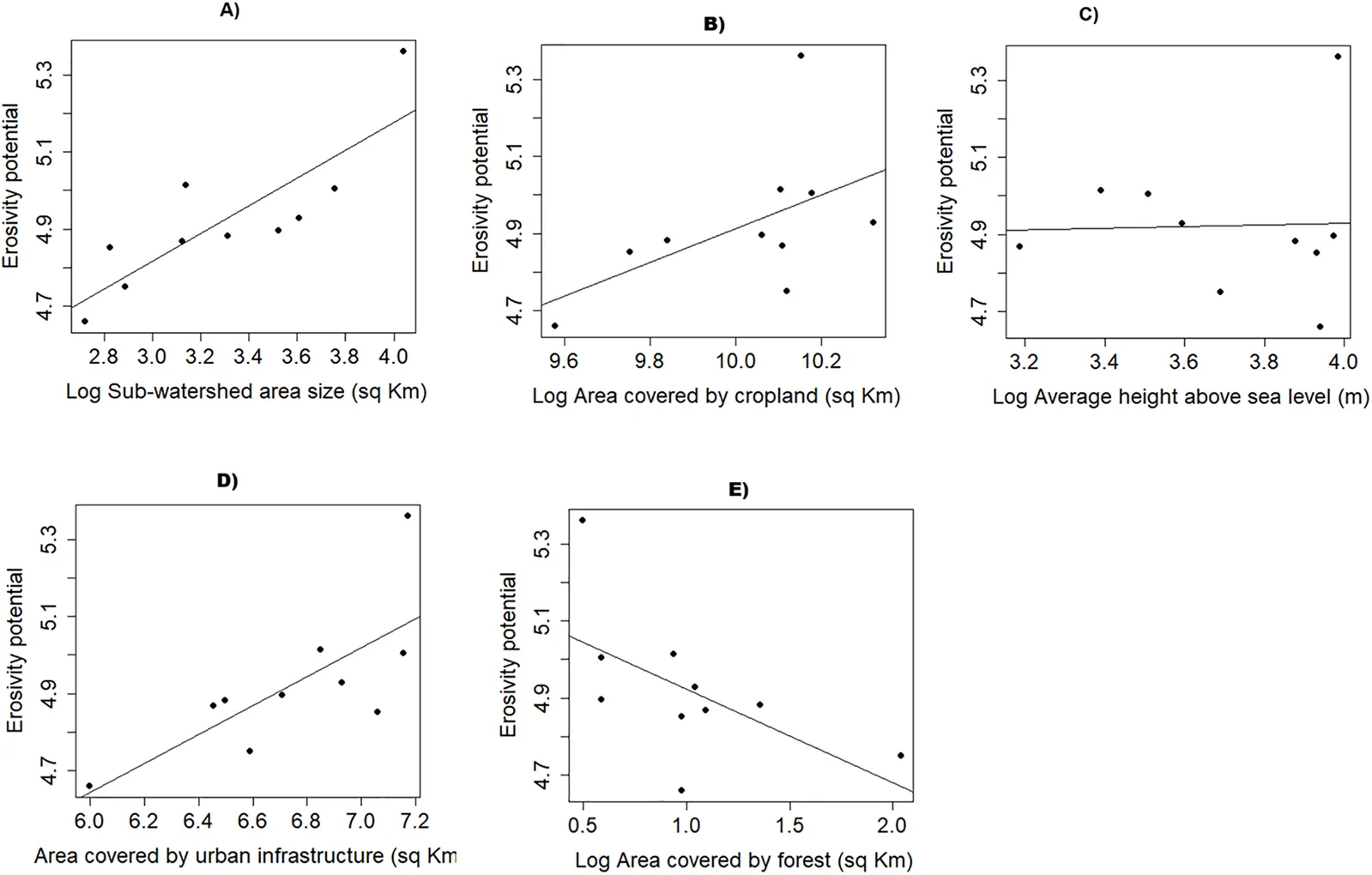
Regression plots of the predicted trends in overall erosion potential (ErPot) response to A). Sub-watershed area size; B) Area covered by cropland; C) Elevation; D) Urban infrastructure; and E) Total area covered by forest.

## Discussion

### Sub-watershed rankings for erosivity and sediment transport

The scale and magnitude of both sediment transport index (STI) and overall erosivity potential (ErPot) were directly correlated to sub-watershed size, which reflects the substantially dominant of geomorphic factors as seen in the formula, rather than the effects of LULCs, in determining the scale of STI and hence ErPot. Conversely, the influence of sub-watershed size on net erosion indicator (NEI) was less notable. For instance, Busia-North and Rangwe, two of the smallest sub-catchments, ranked higher than two of the largest – Mara and Yala-Upper. Again, as seen in the formula this reflects the influence of LULCs in tempering that of geomorphic parameters [37], especially the potential roles of such land cover features as forests and wetlands in mitigating net erosion. Accordingly, larger watersheds with sounder land-use practices may be less vulnerable to effects of erosion than the case for smaller ones which are subjected to non-favorable land-use forms, in spite of slope gradient size [59,68].

### Predicting major drivers of potential erosivity and sediment transport

#### Net erosion indicator (NEI).

The primary positive driver of net erosion indicator was the proportion of landscape covered by cropland, underscoring the significant role that conversion of natural habitat to agriculture plays in promoting surface erosion. This reflects the widely recognized role of agricultural intensification, its expansion or landscape dominance, in reducing ground vegetation cover, and promoting enhanced vulnerability to soil erosion processes [69–72]. Farming activity through various forms of cultivation, perennial tilling, soil mobilization, crop husbandry and frequent harvesting turnovers are all instrumental in loosening surface material and facilitating its movement downstream through stream channel networks and rivers [73,74]. Gao et al. [75] highlighted the negative implications on soil erosion and health, of poor methods of tillage in general and crop plantings in particular, such as downhill row-cropping. Similarly, multiple cycles of semi-annual crops can significantly enhance general soil exposure leading to increased likelihood of sheet and wind erosion [76,77].

Conversely, degree of terrain ruggedness, indicative of general topographic unevenness showed a predicted significant role in reducing the scale of net erosion, suggesting that such unevenness has an important topological role in facilitating retention of eroded material. Iseyemi et al.[78] observed similar trends for the role of agricultural drainage ditches in minimizing transfer of soil and nutrient runoff into downstream river water systems. Proportion of land under forest was similarly shown to be important in mediating reduced net erosion [77] Presence of wetlands was similarly predicted to mitigate the overall scale of net surface erosion, which shows that wetland habitats including dams, ponds, swamps or bogs have a significant potential for trapping and absorbing eroded material thereby potentially reducing volumes that reach the lake. In a study in a coastal plain by Goldman and Needleman [79], presence and cover sizes of wetland habitats were shown to have significant effects in reducing not only the magnitude of erosion but also quantities of agricultural-nutrient surface runoff. Wetlands may not only reduce mobility of small-sized loose material but also process and retain it in form of richer soil and in the process, expand itself and therefore enhance its own capacity to trap further inputs or larger material [80–82].

#### Sediment transport index.

On the other hand, sediment transportation alone was essentially boosted by geological and hydrological factors, basically slope gradient and upslope area size of sub-watershed, no important enhancing roles for crop cover or urban expansion. This finding is in tandem with the well-recognized direct relationship between upslope area size and surface erosivity through its effect on volume of surface runoff and quantity of eroded material [83–85]. As pointed out earlier, this may be indicative of the contrasting significance of LULCs on one hand versus geomorphic and hydrological factors on the other hand, in influencing NEI and STI, respectively [37]. This finding has the implication that the overall load of sediment deposited in the lake originate from multiple sources, with agricultural and urban land being just two of these.

On the other hand, sizes of forest or wetland habitat cover were the two factors predicted to mitigate STI magnitude, with terrain ruggedness playing no part. This observation underscores the important fact that a large sub-watershed may facilitate considerable erosion and transportation of sediment down a steep-sloping landscape which bears no significant vegetation cover or wetland habitat, despite roughness of the terrain. It also means that forests and wetlands are as important in supporting reduced sediment movement as they are in mitigating net surface erosion. Not only do forest tree roots help in binding soil to make it less erodible, their leaves and branch networks also cushion the impact of rainfall and other forms of precipitation to enhance water absorption thus reducing intensity of erosion and mass transportation of eroded sediments from flooding events [86,87]. It can also facilitate reduction in extent or rill or sheet erosion even if the surface soils have been recently loosened, as was shown by Quian et al.[83]. Furthermore, forests and large tree-stands help in breaking the force of non-water related erosion and sediment transport such as by wind or geomorphic processes like landslides, tremors and urban or road construction [88,89].

This is because such parameters may be instrumental in reducing overall sediment connectivity which is typically facilitated through the existing stream channel networks [90–92].

#### Overall erosivity potential (ErPot).

Like STI alone, the combination of net erosion indicator and sediment transport index also, showed a predicted positive response to sub-watershed area size and crop cover size as well as urban infrastructure and elevation. This further concretizes the overall negative implications of agricultural activity and potential expansion of urban areas within lake catchment zones. The importance of forests in potentially redressing the challenges of erosion and sediment transport likelihood or scale was also reaffirmed..The implication here is that the influence of urbanization and agricultural activity in facilitating higher scales of overall erosion and sediment transport down a large watershed, may actually be greater at higher altitudes presumably due to enhanced gravity unless there is a sufficient threshold level of vegetation cover across the landscape Urban development activities such as construction or demolition of buildings and other infrastructure including roads, dams, bridges, airports and stadia can be a significant source of sediment and eroded material moving into water bodies and reservoirs, both in the immediate neighborhood as well as further away through municipal drainage tunnels but also via the connecting network of streams and rivers [18].

### Zonal prioritization and focus for catchment restoration and management

The observed positive linkage between overall erosivity potential and cropland size or urban development on the one hand, and negative linkage to forest cover, on the other, is reflected in the identities of the sub-watersheds that ranked highest in potential erosivity. Specifically, it reflects the fact that for each of the sub-watersheds, there is an interplay between upslope-area size and comparative dominance of land-sue-land-cover elements on one hand, and geomorphic or topographic factors on the other. Thus, smaller sub-watersheds with relatively high crop intensification but lower forest and wetland cover may be more vulnerable to erosion and sediment transport than those larger sub-watersheds with comparatively larger areas covered with forest and wetland habitat. Accordingly, for net erosion indicator to be reduced, catchment rehabilitation effort should best be focused on the Busia-North, Rangwe, Homabay-Noth and Nzoia sub-catchments. By comparison, sediment transport indices and overall erosivity potential showed direct and straight forward correlation to sub-catchment area size Therefore, the highest overall prioritization for staving off STI and ErPot should be focused on the larger sub-watersheds namely the Mara and the Yala zones and the Nzoia.

## Conclusion and recommendations

In general, LULC parameters were comparatively more impactful for erosion and sediment transport processes as compared to topographic and geomorphic factors. Specifically, agricultural activity (specifically crop cover size) and urbanization, were was the most important cross-cutting driver parameter for erosion and sediment transport, whereas extent of landscape cover with forest cover or wetland habitat, were most important mitigating factors [79,82,88]. In this regard, deliberate steps to conserve existing forest stands or to increase forest cover throughout the catchment area is a particularly urgent necessity given the current rapid trends in deforestation across all parts of Kenya and especially in the western regions where this study was conducted [93–95]. On the other hand, the most important topographic enabling factors for erosion and potential sediment deposition are slope gradient and elevation, although terrain unevenness can have important mitigating effects on general erosivity. Therefore, for the sustainable integrity of the Kenyan side of Lake Victoria catchment, conservation, management and restoration effort should prioritize on increasing overall tree cover including through reforestation of degraded forests and on-farm agroforestry [37,42]. Action should also be focused on minimizing farming activity on steep slopes [68,87,96] while also revitalizing efforts to conserve existing networks of wetland habitats and possibly creating new ones [81,97]. These results are readily applicable in a broader sense as a foundation for trend-monitoring initiatives on erosion and sediment transport processes, and controlling key LULC linkages towards staving off the rate of deposition across the Lake Victoria region as a whole, and in similar tropical lacustrine habitats worldwide.

## Supporting information

S1 Fig Results of model fit diagnostic tests, indicating observed Quantile-Quantile and DHARMa residual plots for data distribution, (KS), dispersion and outliers against expected scenarios, for the three response variables A) The fit for the net erosion indicator – NEI; B) The fit for sediment transport index – STI; and C) overall erosivity potential – ErPot. (TIF)

S2 Fig Variance Inflation Factor (VIF) test results for variables retained after selecting best models. (TIF)

S1 File List of the best models selected with the respective retained variables for regression modelling. (NOTE: log = logarithm to base 10; *tri* = terrain ruggedness index; *slope* = mean slope gradient; *elev* = mean elevation; forestcover = total aarea covered by non-plantation forest; *cropcover* = total area covered by cropland; *wetlandcover;* total area covered by wetland habitat; *urbancover* = total area covered by urban infrastructure;).
(PDF)

S2 File Table detailing types, formats and sources of datasets used in the study. (PDF)
