# Supplementary material for: Using GIS, remote-sensing and linear modeling to prioritize spatially explicit action against erosion and sediment transport within Lake Victoria watershed in Kenya
Source: PLoS One. 2026 Oct 1;21(10):e0359542. doi: 10.1371/journal.pone.0359542 (PMC13630186; doi:10.1371/journal.pone.0359542)
Supplement: Supplementary file 1 [file pone.0359542.s003.pdf]

Supporting material Table: Summary description of type and source of datasets used in the analyses

Dataset	Format	Resolution/Accuracy	CRS	Source
Satellite image of Lake Victoria catchment region (Kenyan territory)	Landsat remote-sensed L1 (6 scene panels) 2025 October	30-m (2 meter accuracy)	EPSG: 32637 (WGS84) – after reprojection	Earth Explorer® (USGS)
Kenya counties layer (with market centers	Vector (polygon and points)	• Determined from a 5-yearlong data gathering<br>• Small seasonal markets not included	EPSG: 32637 (WGS84)	World Resources Institute<br>Kenya Bureau of Statistics
Forests and Wetlands cover	Vector (polygons)	Countrywide, and per county	EPSG: 32637 (WGS84)	World Resources Institute
Open street map	Vector	Depends on source and timing (November 2024)	EPSG: 3857 (WGS84/ Pseudo Mercator)	https://tile.openstreetmap.org/{z}/{x}/{y}.png
Elevation; Slope	Raster	5-meter	EPSG: 3857 (WGS84/ Pseudo Mercator)	GoogleEarth Geo
Kenya crops mask	Raster file	Countrywide, and per county	EPSG: 32837 (WGS84)	Regional Center for Mapping Of Resources for Development, Nairobi
