# Supplementary material for: Using GIS, remote-sensing and linear modeling to prioritize spatially explicit action against erosion and sediment transport within Lake Victoria watershed in Kenya
Source: PLoS One. 2026 Oct 1;21(10):e0359542. doi: 10.1371/journal.pone.0359542 (PMC13630186; doi:10.1371/journal.pone.0359542)
Supplement: Supplementary file 2 [file pone.0359542.s004.pdf]

S1 File: List of the best models selected with the respective retained variables for regression modelling.(NOTE: log=logarithm to base 10; *tri*= terrain ruggedness index; *slope*=mean slope gradient; *elev*= mean elevation; forestcover=total aarea covered by non-plantation forest; *cropcover*=total area covered by cropland; *wetlandcover;* total area covered by wetland habitat; *urbancover*=total area covered by urban infrastructure;)

1. Net erosion indicator (NEI)

modNEI<-glmm(logeros~ +logmeanelevation+logTRI+logmeanslope+logforestcover+logcropcover+logwetlandcover+logUrbancover(1|subwatershedID), family = beta, link – logit, data=eros)

2. Sediment transport index (STI)

modSEDIMENT<-glmm(STI~logsub-logmeantri+logmeanslope+logforestcover+logcropcover+logwetlandcover+logurbancover+ watershedarea+logwetlandcover(1|sub-watershedID), family = beta, link – logit, data=sed)

3. Erosivity potential (ErPoT) = NEI+STI

modErPot<-glmm(logerosandsti~logSubwatershedarea+logTRI+logMeanelevation+logForestcover+logCropcover+ logUrbancover, family = beta, link – logit, data=erossti)
