# Supplementary material for: Using GIS, remote-sensing and linear modeling to prioritize spatially explicit action against erosion and sediment transport within Lake Victoria watershed in Kenya
Source: PLoS One. 2026 Oct 1;21(10):e0359542. doi: 10.1371/journal.pone.0359542 (PMC13630186; doi:10.1371/journal.pone.0359542)
Supplement: Supplementary file 3 [file pone.0359542.s005.pdf]

PLOS One

Using linear modeling and remote sensing to identify priority spatial and parameter-based mitigation measures against erosion and sediment movement within Lake Victoria watershed in Kenya

--Manuscript Draft--

**Manuscript Number:**	PONE-D-26-12960
**Article Type:**	Research Article
**Full Title:**	Using linear modeling and remote sensing to identify priority spatial and parameter-based mitigation measures against erosion and sediment movement within Lake Victoria watershed in Kenya
**Short Title:**	Modeling watershed erosion and sedimentation of tropical lake
**Corresponding Author:**	Nickson Erick Otieno, PhD<br>National Museums of Kenya<br>Nairobi, KENYA
**Keywords:**	Hydrology, sedimentation, watershed management, spatial modeling, priority zoning
**Abstract:**	Watershed degradation due to human encroachment is a global phenomenon worldwide which is a keystone driver of erosion, transport and deposition of sediment material into downstream lakes and other water bodies. Despite the wide recognition of these trends, comprehensive evaluation of relative prominence of the different factors driving catchment destruction and subsequent sediment movement, or contributing to its mitigation, has rarely been undertaken. This study applied GIS with remote-sensed Landsat satellite imagery and generalized linear modeling to assess relative significance of an array of topographic, geomorphic and hydrological parameters (slope angle, elevation, terrain ruggedness, stream channel network sizes) as well as land-use land-cover (LULC) elements (crop cover, forest cover, wetland cover, urban-features cover), in driving net erosion indicator (NEI), sediment transport indices (STI), and index of overall erosion potential combining the two (ErPot) across 19 counties forming the watershed of Lake Victoria on the Kenyan territory side. Across the 6 sub-catchments delineated, net erosion indicator was mainly enhanced by size of crop cover, but was mitigated by terrain ruggedness, whereas sediment transport index was predicted to increase with elevation and slope gradient, but to decrease with total cover of forests and wetland habitats. The combined erosion potential (ErPot) was also predicted to be promoted by crop cover size as well as total area covered by urban infrastructure, but also reduced by wetland habitat cover. Thus, regulating agricultural activity or expansion, especially on steep slopes, as well as increasing landscape vegetation cover especially through afforestation and agroforestry initiatives, and wetland conservation, would be the key strategies for sustainable preservation of the lake's watershed. Using these results, the counties of Naymira, Kisii, Kisumu. Kericho and Busia were identified as those of highest priority for potential action focus in efforts to promote restoration and conservation of the lake's catchment.
**Order of Authors:**	Nickson Erick Otieno, PhD
**Additional Information:**	
**Question**	**Response**
**Financial Disclosure**<br><br>Enter a financial disclosure statement that describes the sources of funding for the work included in this submission. Review the submission guidelines for detailed requirements. View published research articles from *PLOS ONE* for specific examples.	The author(s) received no specific funding for this work.

Powered by Editorial Manager® and ProduXion Manager® from Aries Systems Corporation

This statement is required for submission and **will appear in the published article** if the submission is accepted. Please make sure it is accurate.<br><br>**Funded studies**<br>Enter a statement with the following details:<br>• Initials of the authors who received each award<br>• Grant numbers awarded to each author<br>• The full name of each funder<br>• URL of each funder website<br>• Did the sponsors or funders play any role in the study design, data collection and analysis, decision to publish, or preparation of the manuscript?<br><br>Did you receive funding for this work?	
**Competing Interests**<br><br>Use the instructions below to enter a competing interest statement for this submission. On behalf of all authors, disclose any competing interests that could be perceived to bias this work—acknowledging all financial support and any other relevant financial or non-financial competing interests.<br><br>This statement is required for submission and **will appear in the published article** if the submission is accepted. Please make sure it is accurate and that any funding sources listed in your Funding Information later in the submission form are also declared in your Financial Disclosure statement.<br><br>View published research articles from *PLOS ONE* for specific examples.	The authors have declared that no competing interests exist

**NO authors have competing interests**<br><br>Enter: *The authors have declared that no competing interests exist.*<br><br>**Authors with competing interests**<br><br>Enter competing interest details beginning with this statement:<br><br>*I have read the journal's policy and the authors of this manuscript have the following competing interests: [insert competing interests here]*<br><br>* typeset	
**Ethics Statement**<br><br>Enter an ethics statement for this submission. This statement is required if the study involved:<br><br>• Human participants<br>• Human specimens or tissue<br>• Vertebrate animals or cephalopods<br>• Vertebrate embryos or tissues<br>• Field research<br><br>Write "N/A" if the submission does not require an ethics statement.<br><br>General guidance is provided below. Consult the submission guidelines for detailed instructions. **Make sure that all information entered here is included in the Methods section of the manuscript.**	N/A

Powered by Editorial Manager® and ProduXion Manager® from Aries Systems Corporation

**Format for specific study types**<br><br>**Human Subject Research (involving human participants and/or tissue)**<br>• Give the name of the institutional review board or ethics committee that approved the study<br>• Include the approval number and/or a statement indicating approval of this research<br>• Indicate the form of consent obtained (written/oral) or the reason that consent was not obtained (e.g. the data were analyzed anonymously)<br><br>**Animal Research (involving vertebrate animals, embryos or tissues)**<br>• Provide the name of the Institutional Animal Care and Use Committee (IACUC) or other relevant ethics board that reviewed the study protocol, and indicate whether they approved this research or granted a formal waiver of ethical approval<br>• Include an approval number if one was obtained<br>• If the study involved *non-human primates*, add *additional details* about animal welfare and steps taken to ameliorate suffering<br>• If anesthesia, euthanasia, or any kind of animal sacrifice is part of the study, include briefly which substances and/or methods were applied<br><br>**Field Research**<br><br>Include the following details if this study involves the collection of plant, animal, or other materials from a natural setting:<br>• Field permit number<br>• Name of the institution or relevant body that granted permission	
**Data Availability**<br><br>Authors are required to make all data underlying the findings described fully available, without restriction, and from the time of publication. PLOS allows rare exceptions to address legal and ethical concerns. See the PLOS Data Policy and FAQ for detailed information.	Yes - all data are fully available without restriction

Powered by Editorial Manager® and ProduXion Manager® from Aries Systems Corporation

A Data Availability Statement describing where the data can be found is required at submission. Your answers to this question constitute the Data Availability Statement and **will be published in the article**, if accepted.<br><br>**Important:** Stating 'data available on request from the author' is not sufficient. If your data are only available upon request, select 'No' for the first question and explain your exceptional situation in the text box.<br><br>Do the authors confirm that all data underlying the findings described in their manuscript are fully available without restriction?	
**Describe where the data may be found in full sentences. If you are copying our sample text, replace any instances of XXX with the appropriate details.**<br><br>• If the data are **held or will be held in a public repository**, include URLs, accession numbers or DOIs. If this information will only be available after acceptance, indicate this by ticking the box below. For example: *All XXX files are available from the XXX database (accession number(s) XXX, XXX.).*<br>• If the data are all contained **within the manuscript and/or Supporting Information files**, enter the following: *All relevant data are within the manuscript and its Supporting Information files.*<br>• If neither of these applies but you are able to provide **details of access elsewhere**, with or without limitations, please do so. For example:<br><br>*Data cannot be shared publicly because of [XXX]. Data are available from the XXX Institutional Data Access / Ethics Committee (contact via XXX) for researchers who meet the criteria for access to confidential data.*<br><br>*The data underlying the results presented in the study are available from (include the name of the third party*	All relevant data are within the manuscript and its Supporting Information files.

Powered by Editorial Manager® and ProduXion Manager® from Aries Systems Corporation

and contact information or URL).<br>• This text is appropriate if the data are owned by a third party and authors do not have permission to share the data.<br><br>* typeset	
Additional data availability information:	

Powered by Editorial Manager® and ProduXion Manager® from Aries Systems Corporation

Manuscript
Click here to access/download;Manuscript;Revised_MS_CLEAN.docx

Using linear modeling and remote sensing to identify priority spatial and parameter-based mitigation measures against erosion and sediment movement within Lake Victoria watershed in Kenya

Nickson Erick Otieno
National Museums of Kenya, Zoology Department, P. O. Box 50361-00100 Nairobi - Kenya
E-mail: neotieno@yahoo.com Tel +254 726797884

Short title: Modeling watershed erosion and sedimentation of tropical lake

Abstract

Watershed degradation due to human encroachment is a global phenomenon worldwide which is a keystone driver of erosion, transport and deposition of sediment material into downstream lakes and other water bodies. Despite the wide recognition of these trends, comprehensive evaluation of relative prominence of the different factors driving catchment destruction and subsequent sediment movement, or contributing to its mitigation, has rarely been undertaken. This study applied GIS with remote-sensed Landsat satellite imagery and generalized linear modeling to assess relative significance of an array of topographic, geomorphic and hydrological parameters (slope angle, elevation, terrain ruggedness, stream channel network sizes) as well as land-use land-cover (LULC) elements (crop cover, forest cover, wetland cover, urban-features cover), in driving net erosion indicator (NEI), sediment transport indices (STI), and index of overall erosion potential combining the two (ErPot) across 19 counties forming the watershed of Lake Victoria on the Kenyan territory side. Across the 6 sub-catchments delineated, net erosion indicator was mainly enhanced by size of crop cover, but was mitigated by terrain ruggedness, whereas sediment transport index was predicted to increase with elevation and slope gradient, but to decrease with total cover of forests

and wetland habitats. The combined erosion potential (ErPot) was also predicted to be promoted by crop cover size as well as total area covered by urban infrastructure, but also reduced by wetland habitat cover. Using these results, the counties of Naymira, Kisii, Kisumu, Kericho and Busia were identified as those of highest priority for potential action focus in efforts to promote restoration and conservation of the lake's catchment. Thus, regulating agricultural activity or expansion, especially on steep slopes, as well as increasing landscape vegetation cover especially through afforestation and agroforestry initiatives, and wetland conservation, would be the key strategies for sustainable preservation of the lake's watershed. The results are therefore practically applicable in decision making for prioritizing county-based regions for focusing watershed conservation and rehabilitation of degraded zones,

Key words: Hydrology, sedimentation, watershed management, spatial modeling, priority zoning

Introduction

The growing global demand for food due to escalating human populations continues to put pressure on land-based agricultural systems which, however, face increasing challenges relating to dwindling soil qualities, high costs of production and changing weather patterns[1, 2, 3, 4]. This has occasioned an increasing shift in food production and supply chains towards aquatic-based resources to bridge gaps in demands at local, regional and global levels[5, 6]. At the same time, due to water scarcity, more urban residents and industries are relocating to areas closer to more permanent water sources[7, 8, 9]. In low-income economies, especially in tropical regions, this mainly takes the form of populations moving closer to major water bodies such as lakes, dams, rivers and waterways[10, 11]. Such encroachment, reclamation or conversion of riparian, catchments or watershed habitat often leads to significant risks of domestic, agricultural and industrial

pollution but also siltation, excessive abstraction[12, 13] as well as overexploitation of fisheries and other aquatic resources[14, 15].

In particular, agricultural activity on riparian land or catchment zones is often a source of agrochemical runoffs and silt deposition into surface and undergriound water reservours through streams or rivers and even sippage through the soil[16, 17]. Urban development and human settlement on these areas is similarly associated with solid and liquid effluent pollutants from domestic, industrial, construction and demolition activities[18]. The impacts of these activities tend to be significantly enhanced with increased slope gradient[19] and proximity to the water body itself such that pollutants and sediments have more limited chance of being absorbed, diluted or otherwise buffered off before entering the water bodies[20; 21]. Such impacts are further exacerbated by various forms of impacts of climate change[4, 22 including frequent flush floods and fluctuations in lake levels. Additional consequent impacts such as influx of invasive species, reduction in water quality and overall health of organisms[5, 7, 23, 24], all combine to have negative implications for aquatic food webs[25, 26].

Although it is primarily a natural process [27, 28, 29] and is important in supporting general nutrient cycling and distribution[30], accelerated surface erosion or sediment transportation and deposition may be profoundly exacerbated by a wide variety of anthropogenic activities and land-use land-cover (LULC) forms. The effects may be immediate for activities along the shoreline and riparia, but may be longer-term from activities in the catchment zones further upstream[31].

On a global scale, sediment transportation and general surface erosion is mainly attributed to vegetation removal and agricultural activities, typically characterized by frequent vegetation clearing and soil loosening. Industrial and urban developmentare also major factors, especially in highly developed economies. Among its negative impacts are increased risks of coastal flooding,

poor quality of drinking water, reduced health of aquatic species, diminished fisheries and reduced efficiency in maritime transport activities[27, 34]. Regardless of the specific source route, sediment erosion and movement rate or volumes can be significantly accentuated by increased slope gradient especially at higher altitudes[19, 33, 35]. A simulated study by Hatono et al.[32] estimated the global sediment transport phenomenon from catchment to be in the range of 3.0 to 4.6 billion tons annually. Nonetheless, such sediment removal and transport may also be considerably cushioned by rougher terrain, or buffered by enhanced vegetation cover or wetland habitats which serve to either reduce sediment size, per capita amount, or movement speed[33, 36, 37].

Lake Victoria, the second largest freshwater lake in the world and the largest in the tropics[38], supports an estimated population of 42 million people who depend on it as a primary livelihood source in terms of food, water, employment and other goods or services[39, 40]. Fishing activities constitute the main food source but also a key source of revenue for exchequers of the three East African states, but the rapidly growing dependency on the lake's fisheries resources has resulted in considerable overfishing[41, 42]. At the same time, the lake continues to be impacted by other human activities in and around it, including pollution, invasive animal and plant species and rapid riparian development activities[43]. Further away, the catchment continues to be degraded through deforestation, agricultural expansion, human encroachment, urbanization and excessive water abstraction for irrigation, resulting in increased silt loading, further pollution and eutrophication from agricultural, domestic and industrial chemical runoffs[12, 13]. The consequent degradation of the lake, compounded with climate change, is associated with reducing lake levels, water quality and poor health of fisheries, frequently reported in form of significantly declining trends in catches per capita[44, 45]

Majority of past studies assessing sediment transport index [STI] across landscapes tend to examine this parameter in isolation, based incorporation of only two key factors: a) size of the contributing area, and b) slope angle. Only a few studies have consider evaluating the roles of at least one LULC parameter that might potentially enhance or mitigate STI, [19, 20; 21, 37]. Therefore, this study is novel because it is the first one to assess STI comprehensively with the broad context of modeling (rather than spot-mapping) its contribution to overall potential for surface erosion in combination with net surface erosion, in response to a multiplicity of topographic and LULC drivers and mitigating parameters, with specific reference to its impact on a large cross-border tropical freshwater ecosystem. Furthermore, this study area forms part of the wider African Great Lakes and East Rift Valley system considered by UNESCO as a regional hotspots for erosion and sediment transportation, making the results more significant for potential adoption or practical application in national or regional environmental, hydrological or strategic economic planning.

The overall aim of this study was to apply geospatial analysis and linear modeling to evaluate relative influence of a range of topographic and LULC factors that promote or mitigate the scale of surface erosion and sediment movement across 19 administrative counties within the main catchments of Lake Victoria on the Kenyan territorial side, for the purpose of identifying priority zones in which to focus potential efforts for actions that promote sustainable management of the lake's watershed. The specific objectives were:

1. Establish a classification of Lake Victoria catchment area based on county-level ranking of surface erosion, sediment transport indices, and a combination of both;
2. Create predictive linear models for key geomorphic/topographic and LULC parameters driving net surface erosivity, sediment transportation, and a combination of both

3. Highlight the most important geomorphic and LULC parameters that drive or mitigate overall erosivity potential to guide action-based prioritization of sustainable catchment management effort

The general expectation was to find that, when all geomorphic and LULC parameters are kept constant:

a) Counties covered by the larger proportions of catchment upslopes would contribute more to the scale of overall erosion and sediment transportation, owing primarily their correspondingly larger contributing surfaces;

b) Counties or catchment zones closer to the lake would be associated with higher scales of overall erosion and sediment transportation than those further upstream, due to shorter distances for moving sediment material

Methodology

Study area

Lake Victoria is the largest freshwater lake in the world, straddling the 3 east African states of Kenya, Uganda and Tanzania (Fig 1). It covers a total area of 68.800 Km^2, of which 6%, 45% and 49% lie in Kenya, Uganda and Tanzania, respectively[46] with depth ranging from 0-80 m, and averaging 40 m[47]. On the Kenyan side, the lake is surrounded by several urban areas of varying sizes and human populations, the main ones being the city of Kisumu and the towns of Homa Bay, Siaya, Busia and Migori (Fig 1)[48]. The study focused on 19 counties of the western Kenya region covering 01°33´S-01°7´N and 33°58´E-38°10´E, an area of some 51,200 km^2[49] and which

constitute the main watershed of Lake Victoria on the Kenyan territory (Fig 1). This is a large and diverse area topographically as well as climatically, with mean elevations ranging from 1,900-13,400 m above sea level, a mean annual rainfall range between 900 – 2,200 mm, mean annual temperatures between 14° C around Mt Elgon region in Bungoma County to 32° C in parts of Keiyo-Marakwet County[50], and therefore the constituent counties do not necessarily share much environmental uniformity in any broad sense. Several of the extensively hilly, mountainous and other highly-elevated areas within this region constitute the western-Kenya zone of the country's most strategically important natural water sources, also known as Water Towers (KNBS, 2023).

Fig 1. Map of study area indicating the focal area in Kenyan side of Lake Victoria encompassing the counties which form the catchment of the lake (Courtesy: Google Maps, 2026).

Spatial data acquisition and processing

Spatial data were obtained from diverse sources including the Kenya Meteorological Department, the Word Resources Institute Open Data (majority of the shapefiles), the Kenyan National Bureaus of Statistics (KNBS), the East African Regional Center for Mapping OF Resources for Development, RCMRD (the Kenya crop-cover mask layer), the county Government of Kisumu, Friends of Lake Victoria (OSIENALA) and Google Maps archives. All data were processed on a quantum geographical information systems (QGIS), v. 3/4/8) platform. Satellite imagery data were acquired as Landsat 8 Level 1 imagery of land cover data from *Earth Explorer*[51]. Multiple panels of the available datasets were compsited into one mosaic raster layer matching the extent of the selected study region of the Lake Victoria watershed (Fig 2 A, Fig 2B, Fig 2C). From a 90-m spatial resolution srtm digital elevation model [DEM] of Kenya, a clipping was conducted, masking with a vector layer of Kenya counties, labeled accordingly for each County. Using the QGIS's SAGA 9.2 add-on processing tool, the hydrological and terrain analysis functions were

applied in filling sinks in the clipped DEM, and in establishing steam channel networks (Fig 2D) and to identifying major confluents of rivers with the points where they enter into Lake Victoria [Fig 2E]. The clipped layers were subsequently re-projected from Lat/Long coordinate reference system to UTM zone 37N for Kenya (Fig 2E, Fig 2F).

Fig 2. Map of the selected study are indicating: The mosaicked separate panels of the satellite imagery in A) Pseudo-color and B) Grey-scale; C) Indicating the constituent counties on hillshade; D) Showing the entire stream channel network; E) Showing major rivers entering Lake Victoria; and F) Showing the all major rivers network and counties in re-projected form.

The combination of stream channels and major rivers were therefore used for delineating the main catchment zones, and in calculating the respective upslope area sizes. The individually delineated catchment zones and upslope area sizes were then vectorized and combined using the merge procedure, into one vector layer. The merged upslopes were then dissolved to consolidate the total upslope areas size for the entire watershed, before using join by attribute (summation) and zonal statistics to derive total upslope sizes for individual counties, using the Kenya counties map as an overlay vector. A similar process of clipping and masking was employed in processing an additional array of complimentary datasets with respective additional attributes for each of the counties. These additional attributes included vector layers comprising data on areas covered by natural and plantation forest; area covered by cropland' area covered by urban development features and infrastructure including road networks; and area covered by wetland and swamp habitat (excluding rivers). The second set of raster datasets of the clipped and re-projected 90-m spatial resolution srtm DEM and hillshade, was used to derive data on slope gradient, terrain ruggedness indices (TRI) and general elevation for each county[52, 53].

Statistical analyses

Net erosion indicator (NEI)

The fundamental physical and ecological role of each of the key selected topographical and LULC parameters was taken into consideration in calculating the rank value of each county for its role in contribution to overall erosion towards Lake Victoria. This role rank was determined as the net erosion indicator (NEI) which is fundamentally attributable to movement of surface soil and other suspended sediment material by water through the network of stream channels and associated rivers. In this study, it was established as the ratio of the key parameters that primarily promote surface erosion, to those parameters that potentially serve to mitigate erosion through retention of eroded material[19; 37, 54, 55]. Thjs function was expressed in the relationship:

$$NEI = [TUA+Slope+Elev+Cropsize+Urban]/[TRI+Forest+Wetland]$$

Where *NEI* = net erosion indicator; *TUA* = total area of the county overlapping the upslope areas of the six major sub-catchments delineated; *Slope* = Mean slope gradient; *Elev* = mean elevation; *Cropsize* = total size of area covered by crop;based agriculture; *Urban* = total area covered by urban development and other infrastructure; *TRI* = terrain ruggedness index; Forest = total size of area covered by perennial woody plants; *Wetland* = percent area covered by wetland habitat. Prior to application of the formula, data were standardized through re-scaling by use of logarithmic transformation[56]**.** The selection of this range of topographic and LULC parameters for determining net erosion indicator was guided by the range of variables outlined by the United Nations Educational, Scientific and Cultural Organization (UNESCO) International Sediment Initiative's assessment report on key drivers of erosion and sediment movement across regions of the world that are regarded as the global erosion hotspots[33].

Sediment Transport Index (STI)

For each county, the sediment transport index (the downward movement of soil particles and other material of various sizes[36, 57], with or without the agency of water, including other geomorphic processes that loosen the earth surface and trigger downward movement of suspended and non-suspended material under gravity, such as landslides, tremors, cultivation, construction or demolition activity) was calculated from the relationship:

$$STI = [A / 22.13]^{0.6} * [\sin[\beta] / 0.0896]^{1.3}$$

where '*A*' = the contributing area within the delineated stream channel upslope area (i.e. the region contributing to the upslope per unit contour length); and '***β***' = slope angle or gradient in degrees[58, 59].

Determining overall erosivity potential (ErPot)

The overall erosivity potential for each county was calculated as the combined sum of the net erosion indicator (NEI])and sediment transport index (STI), representing removal and downstream movement of eroded material both by water and by other processes. It was determined from the relationship:

$$ErPot = NEI + STI$$

where *ErPot* = overall potential of the county to contribute to erosion and transportation of eroded material towards Lake Victoria; *NEI* = the net erosion indicator; *STI* – sediment transport index

The

Modeling influence of topographic and LULC factors on erosion and sediment transport

Predictive models were developed for assessing patterns of relationships between the topographic features or LULC parameters on overall potential incidences or severity of net surface erosion (NEI) and sediment transport (STI), both individually and in combination. Here, generalized linear mixed models (GLMM) were used in *R v 4.4.2* programing language within the *lme4* and *multcomp* packages[60, 61]. These were conducted, in successive turns, for NEI, *STI* and then overall erosivity potential (*ErPot = NEI+STI)*. The error distribution family used was beta, with the *logit* link function. In each case, size of county was included as a random factor, due to the observable considerable variations in this variable; while treating the other topographic and LULC elements as fixed effects[61]. Since tests on spatial autocorrelation of the variables did not indicate any significance, county identity was not included as an additional random factor[62].

Before analyses, variables were inspected for multi-colliniarity, using the variance inflation factor (VIF) procedure through the function *vif* from the package *car* [63], setting a maximum VIH threshold of 4. No variables were found to be significantly collinear (S1 Fig). For each case, full models were first fitted with all variables retained after VIF, and then model fit diagnostics were conducted to test for data dispersion using the *glmmTMB* function within the *DHARMa* procedure in the *AER* package, and it was confirmed through plots of quantile-quantile and the rank-transformed DHARMa dispersion residuals (S2 Fig), that there were no significant overdispersions in each case[64 65, 66]. Subsequently, a dredge process was applied within the *MuMln* package to select the best models stepwise through the Akaike Information Criterion (AIC) modified for small samples, setting a delta AIC value at $\Delta AIC \leq 2$[67]. A total of 27 candidate models with multiple combinations were considered, Following the model selection procedure, the best models were those without upslope area, for the case of the NEI and STI as separate response variables, and those without upslope area, terrain ruggedness index, slope angle, elevation and wetland cover, for the

case of NEI+STI (see S1 File for the best models selected with the respective retained variables). Model selection was followed by use of the function *model.avg* to select the best conditional model subset, before evaluating conditional R squares for the remaining model subsets with the *r.squares* function, repeating this for each response variable at a time[67]. All significant effects were evaluated at $p \leq 0.05$.

Ethics approval and informed consent

As the study did not involve any handling of animal or human subjects, no formal ethical approval was required from the Ethics Committee of the National Museums of Kenya or the Kenya Wildlife Service. Informed consent was obtained verbally from relevant representatives of all the respective counties included in this study.

Results

Stream channel network and catchment delineation

A total of six main sub-catchment areas were delineated, constituting the key upslopes areas of various sizes associated with corresponding major rivers draining the Lake Victoria watershed on the Kenyan side. In decreasing rank order of size, these were: the Mara, the Yala-main, the Homabay North, the Gem-Yala, the Nyando-Muhoroni, and the Nzoia (Table 1, Fig 3).

Table 1. Sizes and other general attributes of the size major catchment zones identified as potentially contributing to sediment transport into Lake Victoria

Catchment name	Total upslope area size (km^2)	Main associated river	Number of counties drained
Mara	11,522	Mara	7
Yala (main)	11,044	Yala	7
Homabay North	3,497	Awach	4
Yala-Gem	3,042	Yala	3
Nyando	2,144	Nyando	4
Nzoia	1,447	Nzoia	3

Fig 3. Representation of locations and sizes of the six identified major catchment zones and their associated channel networks potentially contributing to erosion and sediment transport into Lake Victoria

County rankings for NEI, STI and ErPot

The rank profiles for each of the counties in terms of net erosion indicator (NEI), sediment transport index (STI) and overall erosivity potential (ErPot), are as shown in Fig 4, generally demonstrating that the various topographic and LULC forms or parameters contribute in different ways and magnitudes to the overall values of NEI, STI and ErPot. Similarly, these rankings were generally diversified, without any evidence of county size determining the trends (Fig 4A-C). However, in general, whereas counties closer to the lake contributed more towards sediment transportation and overall erosion potential (Fig 4B, Fig 4C), the net erosion component was shared more

widely or equitably across the counties in general (Fig 4A). The top counties contributing to net erosion were Nyamira, Kisii, Kericho, Nandi and Keiyo Marakwet, The top counties in terms of sediment transport loading were Nyamira, Kisii, Kisumu, Siaya and Vihiga, but those highest ranking for overall erosion and sedimentation potential are Nyamira, Kisii, Kisumu, Kericho and Busia, These ranks therefore reflect corresponding county level rank priorities for catchment conservation and restoration.

Fig 4. **County rankings for A) net erosion indicator (NEI); B) sediment transport indices (STI); and C) overall erosivity potential (ErPot).**

Predictive models of topographic and LULC parameter influences

Independent effects of NEI and STI

Whereas the total area covered by crops was predicted to enhance the net erosion indicator index (Table 2, Fig 5 B), degree of terrain unevenness (terrain ruggedness index) and presence of wetland habitat were both predicted to mitigate net erosion across all counties (Table 2, Fig 5 A and C). Catchment size, slope angle, height above sea level, size of area covered by either forest or urban development did not appear to influence net erosion.

Table 2. Results of generalized linear mixed tests for predicted trends of net erosion indicator and sediment transport indices in response to the various scenarios of the topographic and LULC parameter for the final selected models. *TRI*=Terrain ruggedness index; *Slop*e=General slope angle; *Elevation*=Mean elevation.

Response variable	Predictor variable	Parameter estimate	Standard error	Z statistic	p≤0.05	Conditional R square
Net erosion indicator (NEI)	**TRI**	**-0.020**	**0.001**	**-16.201**	**<0.001**	0.981
	Slope	-0.278	0.136	2.037	0.066	
	Elevation	-0.060	0.059	-1.006	0.336	
	Forest cover	0.007	0.033	0.228	0.823	
	**Crop cover**	**1.063**	**0.082**	**12.900**	**<0.001**	
	Urban cover	0.017	0.046	0.365	0.722	
	**Wetland cover**	**-0.599**	**0.146**	**-4.100**	**0.002**	
Sediment transport index (STI)	TRI	-0.033	0.018	-1.816	0.097	0.947
	**Slope**	**20.925**	**2.031**	**10.302**	**<0.001**	
	**Elevation**	**2.434**	**0.885**	**2.749**	**0.018**	
	**Forest cover**	**-1.423**	**0.488**	**-2.914**	**0.014**	
	Crop cover	-0.104	1.226	-0.085	0.934	
	Urban cover	1.001	0.618	1.620	0.136	
	**Wetland cover**	**-9.875**	**3.735**	**-2.644**	**0.017**	

Fig 5. Regression plots of the predicted trends net erosion indicator response to A) Terrain ruggedness, B) total cropland size and C) Total area covered by wetland habitat.

On the other hand, sediment transportation was predicted to be enhanced by higher elevation and slope angles (Table 2; Fig 6 A and B) while it was also mitigated by the extent of forest cover and area covered by wetland habitat (Table 2,; Fig 6 C and D). The other variables did not show any predicted bearing on sediment transport scales.

Fig 6. Regression plots of the predicted trends sediment transport index response to A) Slope size; B) Elevation; C) Area covered by forest; and C) Total area covered by wetland habitat

The effects of erosion potential (ErPOT) – (NEI and STI combined)

The overall potential for erosion and sediment transport, representing the combination of NEI and STI showed a predicted positive response to extent of crop cover and urban development (Table 3; Fig 7A-B), but was predicted to be significantly mitigated only by total area covered by forest (Table 3; Fig 7B).

Table 3. Results of generalized linear mixed tests for predicted trends of overall erosivity potential (ErPot) in response to the various scenarios of the topographic and LULC parameter for the final selected models. *TRI*=Terrain ruggedness index; *Slope*=General slope angle; *Elevation*=Mean elevation.

Predictor parameter	Parameter estimate	Standard error	Z statistic	p≤0.05	Conditional R square
Slope	-0.797	0.903	-0.882	0.392	
**Forest cover**	**-0.540**	**0.195**	**-2.775**	**0.014**	0.525
**Crop cover**	**2.211**	**0.767**	**2.882**	**0.011**	
**Urban cover**	**1.002**	**0.619**	**1.620**	**0.046**	

Fig 7. Regression plots of the predicted trends overall erosion potential (ErPot) response to A) A) Area covered by cropland; and B) Total area covered by forest.

Discussion

County rankings for erosivity and sediment transport

Size of county or catchment area did not emerge as important factors in determining the ranking for contribution to net erosion. For instance, Nyamira and Kisii Counties are two of the smallest but ranked higher than the three largest - Narok, Uasin Gishu or Baringo. The same trend was

observed with regards to sediment transportation and overall erosivity rankings, implying that the size of catchment upslope in the case of net erosion, or the size of contributing area, for the case of sediment transport, may not be the sole driver of counties' contribution to the overall magnitude and scale of material movement into Lake Victoria.

Nonetheless, counties closer to the lake ranked higher than those further away in their potential contribution to sediment transport alone, as well as that of overall erosivity potential. This indicates that proximity to the lake predisposes counties towards greater input of sedimentary material into the lake, presumably because for such shoreward counties, sediment material have a shorter movement distance, with a correspondingly reduced likelihood of being slowed down by land-cover obstacles or other anthropogenic features 55]. Conversely for counties further from the lake, sediment material movement, particularly if not directly through mainstream rivers, may be hampered by a variety of natural or anthropogenic landscape barriers and filters over a longer distance and may therefore not necessarily reach the lake[17, 68, 69, 70]. Furthermore, due to the longer distance, by the time many rivers and streams from far-away counties enter the lake, the progressive reduction in flow rate can facilitate higher deposition likelihood for substantial proportions of the larger-size sediment material on the river's own channel bed[71, 72]. This may include large gravels and small stones as was reported by Wohl et al, [72] and also by Lwanga et al.[73]. In addition, there is a growing national intensity in river sediment material harvesting activity to meet growing demands by the construction industry, and for the case of rivers moving longer distances towards Lake Victoria, this may partly explain the rivers' potential to discharge lower quantities of sediment material [73].

On the other hand, net surface erosion, which is predominantly influenced by water movement as a medium of transport, was not heavily influenced by proximity to the lake. This

implies that for this type of eroded material mainly in form of soil (fine sand, silt and clay and nutrients), the channel network of well-established streams and rivers are the main pathways of movement into the lake. Therefore, there are significantly lower chances of movement hinderance and filtering, regardless of distance travelled, if other man-made and topographic variables are kept constant[59, 70]. Thus, counties as far away from the lake as Nakuru and Baringo may contribute as much to net erosion as Bungoma and Homa Bay which are much closer by.

Predicting major drivers of potential erosivity and sediment transport

The sole positive driver of net erosion indicator was the proportion of landscape covered by cropland, underscoring the significant role that conversion of natural habitat to agriculture plays in promoting surface erosion. This reflects the widely recognized role of agricultural intensification, its expansion or landscape dominance, in reducing ground vegetation cover, and promoting enhanced vulnerability to soil erosion processes[74, 75, 76, 77]. Farming activity through various forms of cultivation, perennial tilling, soil mobilization, crop husbandry and frequent harvesting turnovers are all instrumental in loosening surface soil and facilitating its movement downstream through the network of stream channels and rivers[78, 79]. Gao et al.[80] highlighted the negative implications on soil erosion and health, or poor methods of tillage in general And crop plantings in particular. Multiple cycles of semi-annual crops can significantly enhance general soil exposure leading to increased likelihood of sheet and wind erosion[68, 81].

Conversely, degree of terrain ruggedness, indicative of general topographic unevenness showed a predicted significant role in reducing the scale of net erosion, suggesting that such unevenness has an important topological role in facilitating retention of eroded material. Iseyemi et al.[82] observed similar trends for the role of agricultural drainage ditches in minimizing transfer of soil and nutrient runoff into downstream river water systems. Presence of wetlands similarly served to mitigate the overall predicted scale of net surface erosion, which shows that wetland habitats including dams, ponds, swamps or bogs have a significant potential for trapping eroded material and reducing the volume that reaches the lake. In a study in a coastal plain by Goldman and Needleman[83], presence and cover sizes of wetland habitats were shown to have significant effects in reducing not only the magnitude of erosion but also quantities of agricultural nutrient sediment through surface runoff. Wetlands may not only reduce mobility of small-sized loose material but also process and retain it in form of richer soil, and in the process, expand itself and therefore enhance its own capacity to trap even further inputs of larger material[84, 85, 86].

On the other hand, sediment transportation alone was positively enhanced primarily by slope gradient and elevation, and mitigated by the extent of forest and wetland cover, but with no important roles for crop cover size or terrain ruggedness. This finding has a number of possible implications. Firstly, the overall load of sediment deposited in the lake originate from multiple sources, with agricultural land being only one of these. Secondly, terrain unevenness has a less important role to play in mitigating overall sediment movement, as compared to its significance in reducing surface erosion alone. Thirdly, increased elevation can augment the role of slope gradient in facilitating higher scales and perhaps higher rates of sediment movement downstream. Fourth, wetlands are as important in supporting reduced sediment movement as they are in mitigating net surface erosion, Fifth, improved forest cover may be more important in mitigating sediment

movement than in facilitating reduced surface erosion per se. The latter implication is particularly poignant given the fact that most forest stands usually tend to occur in areas of generally higher elevation on both local- and regional-landscape scales[87]. Not only do forest tree roots help in binding soil to make it less erodible, their leaves and branch networks also cushion the impact of rainfall and other forms of precipitation to enhance water absorption thus reducing intensity of erosion and mass transportation of eroded sediments from flooding events[69, 88]. It can also facilitate reduction in extent or rill or sheet erosion even if the surface soils have been recently loosened, as was shown by Quian et al.[89]. Furthermore, forests and large tree-stands help in breaking the force of non-water related erosion and sediment transport such as by wind or geomorphic processes like landslides, tremors and urban or road construction[90, 91].

Counterintuitively, influence of size of river-catchment upslope on the scale of net erosion and sediment transportation, was not significant. This was contrary to the frequently-reported direct relationship between upslope area size and surface erosivity through its effect on volume of surface runoff and quantity of eroded material[89, 92, 93]. Thus, our finding suggests that the role of upslope area in determining the scale of erosion may be offset by the level of ruggedness of the landscape terrain, as well as presence of wetlands and other LULC forms and topographic features that reduce sediment flow rate and instead promote its retention[55, 90]. This is because such parameters may be instrumental in reducing overall sediment connectivity which is typically facilitated through the existing stream channel networks[95, 96, 97].

Finally, overall potential for erosivity (ErPoT) combining both net erosion indicator and sediment transport index, showed a predicted positive response to crop cover size and urban infrastructure, but a negative response to forest cover size, thus reaffirming the negative implications of unregulated agricultural activity and expansion in catchment zones, while further

underscoring the value of forest cover in redressing the challenges of overall likelihood of surface erosion and sediment movement downstream. Urban development activities such as construction or demolition of buildings and other infrastructure including roads, dams, bridges, airports and stadia can be a significant source of sediment and eroded material moving into water bodies and reservoirs, both in the immediate neighborhood as well as further away through municipal drainage tunnels but also via the connecting network of streams and rivers[18].

Zonal prioritization and focus for catchment restoration and management

The observed positive linkage between overall erosivity potential and cropland size or urban development on the one hand, and negative linkage to forest cover, on the other, is reflected in the identities of the counties that ranked highest in potential erosivity. Specifically, it reflects the counties' relative levels of agricultural intensification and corresponding deficits in forest cover or significant wetland habitat in contributing to this parameter. Thus, despite having many wetlands, Kisumu County has the lowest forest cover by size proportion [98, 99], as well as the highest concentration of urban centers and infrastructure in the region while having a predominantly flat terrain of poorly-drained sandy and black-cotton soils in a low-lying area especially around the lake. Similarly, in spite of its relatively high plantation forest cover by proportion, the Kericho County landscape is predominated by crop farming activity, including in area in close proximity to the few wetlands available, thus undermining the potential buffering effect of such habitat through sediment retention[70, 100]. In addition to Kericho's highly elevated location, most of the subsistence farming activities also occur within steep slopes, further enhancing vulnerability to

soil loss towards downstream regions[101]. For Nyamira and Kisii counties, the problems of low forest cover, high crop cover, high elevation and slope-face farming are further aggravated by the extremely high human population density[48], given that these are the smallest counties in the Lake Victoria catchment region as well as nationally[48,49]. These four counties should therefore be of the highest primary or priority focus for efforts aimed at landscape-scale conservation or habitat restoration for sustainable management of the Lake Victoria watershed.

Conclusion and recommendations

The study has shown that the size of river catchment upslope is not the most important determinant of overall erosion and sediment transportation magnitudes, and that while reduced proximity to the lake may limit counties' contribution to sediment transport into the lake, it serves little purpose in minimizing their contribution to overall erosion. Secondly, from the range of LULC forms, agricultural activity is one of the most significant enablers of general surface erosion and sediment movement whereas extent of landscape cover by forests and wetlands are most important for reducing potential magnitudes of erosion and sediment movement[83, 86, 90]. Increasing forest cover throughout the catchment area is a particularly urgent necessity given the rapid trends in deforestation across all parts of Kenya and especially in the western regions where this study was conducted[98, 99, 102]. On the other hand, the most important topographic enabling factors for erosion and potential sediment deposition are slope gradient and elevation, although terrain unevenness can have a mitigating effect especially for net erosion. Therefore, for the Lake Victoria catchment, in the identified priority counties ranked by highest levels of potential erosivity, management and restoration activities should focus on increasing overall tree cover including through reforestation

of degraded forests and on-farm agroforestry[42 37], but also minimizing farming activity on steep slopes[88, 103, 104] while also revitalizing efforts to conserve existing networks of wetland habitats and possibly creating new ones[85, 105].

Limitations of the study

Although the models in this study predicted and highlighted size of agricultural land, forest and wetland cover as the prime-driver LULC forms for erosion and sediment transport dynamics, it did not account for potential effects of these parameters at all fine scales. In other words, the observed and predicted trends would most efficiently apply at larger spatial scales than at local or house-hold scales. In the particular case of the role of cropland cover, no consideration was given to plot-level cropping and structural farm management systems which may also exert certain levels of influence on likelihood or rates of material removal or movement. For instance, cover-cropping, maintenance of uncultivated margins and hedgerows, as well as establishment of terraces and trenches have been shown to facilitate significant reduction in soil loss and nutrient run-off[81, 69, 17]. Similarly, the benefits of forests in mitigating the scale of erosion and sediment transport are more likely to be realized sustainably for large stands of broad-leaved perennial trees growing in high density, than for smaller-patch matrices of thin-leaved temporary plantation forests[106, 107]. Likewise, most forms of natural and artificial wetlands of medium to large sizes are of great importance in buffering sediment movement or aiding their absorption and retention, but smaller ephemeral wetlands may be less efficient for this role, while certain forms of constructed wetlands such as dams and irrigation water impoundments may increase risk vulnerability of surrounding

landscapes to incidental mass transportation of sediments through disasters arising from structural malfunction or during periods of excessive flooding[108, 109].

Acknowledgements

I am very grateful to the Friends of Lake Victoria [OSIENALA], the Kisumu County Government, the East Africa Regional Centre for Mapping of Resources for Development [RCMRD], the Kenya Meteorological Department, the Kenya National Bureau of Statistics, The Kenya National Council for Population and Development and the World Resources Institute for availing open-source data used as part of the project, and the National Museums of Kenya for co-hosting the project and availing the project management, data analysis infrastructure support.

References

1. Ashraf, J.; Javed, A. (2023). Food Security and Environmental Degradation: Do Institutional Quality and Human Capital Make a Difference? *J. Environ. Manag 331:* 117330
2. Nguyen, T.T.; Grote, U.; Neubacher, F.; Rahut, D.B.; Do, M.H.; Paudel, G.P. Security Risks from Climate Change and Environmental Degradation: Implications for Sustainable Land Use Transformation in the Global South. *Curr. Opin. Environ. Sustain*, *63:* 101322
3. Guiné, R. P. F. (2024). The Challenges and Strategies of Food Security under Global Change. *Foods*, *13*(13): 2083. https://doi.org/10.3390/foods13132083.
4. Hultgren, A., Carleton, T., Delgado, M., Gergel, D., Greenstone, M., Houser, T., Hsiang, M, Rode, A., Yuan, J. (2025). Impacts of climate change on global agriculture accounting for adaptation. Nature. 642: 644-652. https://doi.org/10.1038/s41586-025-09085-w .

5. Brudevoll, A. (2025). Aquatic food in the blue economy – A holistic perspective of drivers, trends, and potential implications for policy and programming. FAO Fisheries and Aquaculture Circular, No. 1279. Rome, FAO. https://doi.org/10.4060/cd5224en
6. Kumar, K. (2025). Aquatic foods in human health, nutrition, and overall welfare. Clinical Nutrition Open Science Volume 64, December 2025, Pages 332-342. https://doi.org/10.1016/j.nutos.2025.11.005
7. Orewole, M, Alaigba, D., Oviasu, O. (2015). Riparian corridors encroachment and flood risk assessment in Ile-Ife: A GIS perspective. Open Journal of Geosciences. 2015. https://doi.org/10.15764/GEOS.2015.01002.
8. Kaushal, S., McDowell, W., Wollheim, W., Newcomer J., Mayer, P., Belt, K., Pennino, M. (2015). Urban evolution: the role of water. Water. 7: 4063-4087. Httpts://doi.org/10.3390/w7084063. 7(8):4063-4087
9. Xiao, W., Chen, W., Yue, W/. Mu, J., Xu, S. (2022). Waterbody loss due to urban expansion of large Chinese cities in last three decades. Scientific Reports. 12. https://doi.org/10.1038/s41598-022-22286-x .
10. Ogutu-Ohwayo, R., Balirwa, J. (2006). Management challenges of freshwater fisheries in Africa. Lakes and Reservoirs: Research and Management. 11. 215 - 226. https://doi.org/10.1111/j.1440-1770.2006.00312.x .
11. Andreasen, M., Agergaard, J., Kofie, R., Møller-Jensen, L., Oteng-Ababio, M. (2022). Urban encroachment in ecologically sensitive areas: drivers, impediments and consequences. Buildings and Cities. 3: 920. https://doi.org/10.5334/bc.210 .

12. Poste, A.E., Muir, D.C.G., Guildford, S.J., Hecky, R.E., 2015. Bioaccumulation and biomagnification of mercury in African lakes: The importance of trophic status. Sci. Total Environ. 506–507, 126– 136. https://doi.org/10.1016/j.scitotenv.2014.10.094

13. Ribbe, N., Arinaitwe, K., Dadi, T., Friese, K., von Tümpling, W., 2021. Trace – element behaviour in sediments of Ugandan part of Lake Victoria : results from sequential extraction and chemometrical evaluation. Environ. Earth Sci. 80, 1–14. https://doi.org/10.1007/s12665-021-09554-1.

14. Jamu, D., Banda, M., Njaya, F., Hecky, R. (2011). Challenges to sustainable management of the lakes of . Lancet. 37: 3-14. https://doi.org/10.1016/j.jglr.2010.11.017 .

15. Odoli, C., Owiti, H., Mwamburi, J., Oduor-Odote, P., Nyaboke, H., Nyamweya, C., Aura, C. (2022). Challenges to the lake fisheries, and factors affecting the effectiveness of a co-management regime in African Great Lakes: A case study of Lake Victoria, Kenya. Aquatic Ecosystem Health and Management. 25:28-36. https://doi.org/10.14321/aehm.025.04.28 .

16. Montgomery, D. R. (2007). Soil erosion and agricultural sustainability. *Proc. Natl. Acad. Sci. U.S.A.* 104:13268–13272 (2007).

17. Juncal, L., Masino, P., Bertone, E., Stewart, R. (2023). Towards nutrient neutrality: a review of agricultural runoff mitigation strategies and the development of a decision-making framework. Science of The Total Environment. 874: 162408. https://doi.org/10.1016/j.scitotenv.2023.162408

18. Blaszczak, J., Steele, M., Badgley, B., Heffernan, J., Hobbie, S., Morse, J., Rivers, E., Hall, S., Neill, C., Pataki, D., Groffman, P., Bernhardt, E. (2018). Sediment chemistry of

urban stormwater ponds and controls on denitrification. Ecosphere. 9: e02318. https://doi.org/10.1002/ecs2.2318

19. Zhang K, Xuan W, Yikui B, Xiuquan X (2021). Prediction of sediment transport capacity based on slope gradients and flow discharge. PLoS ONE 16(9): e0256827. https://doi.org/10.1371/journal.pone.0256827

20. António, S., van der Werf, J., Horstman, E., Caceres, I., Alsina, J., van der Zanden, J., Hulscher, S. (2023). Influence of beach slope on morphological changes and sediment transport under irregular waves. Journal of Marine Science and Engineering. 11: 2244. https://doi.org/10.3390/jmse11122244 .

21. Xie, W., Shimozono, T., Tajima, Y.. (2024). Wave-gravity-induced sediment transport on steep shoreface. Coastal Engineering Journal. https://doi.org/10.1080/21664250.2024.2319396

22. Thodsen, H., Hasholt, B., Kjærsgaard, J.. (2008). The influence of climate change on suspended sediment transport in Danish rivers. Hydrological Processes. 22: 764 - 774. https://doi.org/10.1002/hyp.6652.

23. Sun, R., An, D., Lu, W/. Shi, Y., Wang, L., Wang, Q. (2016). Impacts of a flash flood on drinking water quality: Case study of areas most affected by the 2012 Beijing flood. Heliyon. 2: e00071. https://doi.org/10.1016/j.heliyon.2016.e00071.

24. Carpenter, S. R. (2005). Eutrophication of aquatic ecosystems: Bistability and soil phosphorus. *Proc. Natl. Acad. Sci. U.S.A.* 102:10002–10005

25. Donohue, I., Molinos, J. (2009). Impacts of increased sediment loads on the ecology of lakes. Biological reviews of the Cambridge Philosophical Society. 84. 517-31. https://doi.org/10.1111/j.1469-185X.2009.00081.x.

26. David, P.. Thébault, E., Anneville, O., Duyck, Loeuille, N. (2017). Impacts of invasive species on food webs: A Review of Empirical Data. Advances in Ecological Research. 56: 1-60. https://doi.org/10.1016/bs.aecr.2016.10.001.

27. Beusen, A., Dekkers, A. and Bouwman, A., Ludwig, W., Harrison, J. (2005). Estimation of global river transport of sediments and associated particulate C, N, and P. *Global Biogeochemical Cycles*. 19(4): 1-17. https://doi.org/10.1029/2005GB002453.

28. Walling, D. E. (2009). The impact of global change on erosion and sediment transport by rivers: current progress and future challenges. UNESCO World Water Assessment Program – The International Sediment Initiative, Paris. 29 p.

29. Jenny, J., Koirala, S., Gregory-Eaves, I., Carvalais, N. (2019). Human and climate global-scale imprint on sediment transfer during the Holocene. PNAS 116 (46): 22972-22976 https://doi.org/10.1073/pnas.1908179116

30. Kirwan, M. L., Megonigal, J. P. (2013). Tidal wetland stability in the face of human impacts and sea level rise. Nature 504: 53-60.

31. Owens, P., Petticrew, E., Perk, M. (2010). Sediment response to catchment disturbances. Journal of Soils and Sediments. 10: 591-596. https://doi.org/10.1007/s11368-010-0235-1.

32. Hatono, M., Nitta, T., Arakawa, T., Yoshimura, K. (2018). Modeling sediment dynamics at the global scale. Proceedings of the Fall 2018 AGU Meeting. Pp EP21D-2293}. https://ui.adsabs.harvard.edu/abs/2018AGUFMEP21D2293H}.

33. Golosov, T., Walling, D. E. (2019). Erosion and sediment problems: Global hostpots. The United Nations Educational, Scientific and Cultural Organization. Paris

34. Fratini, C., Anselmi, S., Renzi, M. (2025). Dredge sediment as an opportunity: a comprehensive and updated review of beneficial uses in marine, river, and lagoon eco-systems. Environments: 12(6): 200. https://doi.org/10.3390/environments12060200

35. Mir, A. A., Patel, M. (2024). "A comprehensive review on sediment transport, flow dynamics, and hazards in steep channels." Journal of Water Management Modeling 32: C517. https://doi.org/10.14796/JWMM.C517 www.chijournal.org ISSN: 229.

36. Chenguang Liu [a], Suhua Fu [a b c], Zhanbin Li [a b], Zeyu Zhang [a], Jianhui Zeng [a] (2023). Effects of sediment characteristics on the sediment transport capacity of overland flow. International Soil and Water Conservation Research. 11(1): 75-85.

37. Bisimwa, A.M., Hwaba, M., Akuzwe, D., Guesri, B., Muhaya, Kankonda, A.B. (2025). Effects of land use-land cover changes on hydrology and sediment transport in Bukavu urban river catchments, Lake Kivu basin, Democratic Republic of the Congo. J Great Lakes Research 519(6): 102700. https://doi.org/10.1016/j.jglr.2025.102700

38. Okechi, J., Peoples, N., Nyamweya, C., Glaser, S., Kaufman, L. (2022). The ecological health of Lake Victoria (Kenya) in the face of growing cage aquaculture. Conservation Science and Practice. 4(4): 1-12. https://doi.org/10.1111/csp2.12826.

39. UNEP, 2006. Lake Victoria Basin Environment Outlook: Environment and Development. UNEP, Nairobi.

40. Nyamweya, C.S., Nyaboke, H.M., Aura, C.M., Momanyi, K.N., Mlaponi, E., Odoli, C.O., Njiru, J.M., 2023. Lake Victoria's bounty: A case for riparian countries' blue economic investment. Front. Environmental Science 10, 1–6. https://doi.org/10.3389/fenvs.2022.952654

41. Yongo, E., Manyala, J., Agembe, S. (2018). Growth, mortality and recruitment of silver cyprinid (*Rastrineobola argentea*) in the open waters of Lake Victoria, Kenya. Lakes and Reservoirs Research and Management. 23(3): 244-249. https://doi.org/10.1111/lre.12230

42. Nyamweya, C.S., Nyaboke, H.M., Aura, C.M., Momanyi, K.N., Mlaponi, E., Odoli, C.O., Njiru, J.M., 2022. Lake Victoria's bounty: A case for riparian countries' blue economic investment. Front. Environ. Sci. 10, 1–6. https://doi.org/10.3389/fenvs.2022.952654.

43. Mugo, R., Waswa, R., Nyaga, J.W., Ndubi, A., Adams, E.C., Flores-Anderson, A.I., 2020. Quantifying land use land cover changes in the Lake Victoria Basin using satellite remote sensing: the trends and drivers between 1985 and 2014. Remote Sens. (Basel) 12, 2829. https://doi.org/10.3390/rs12172829

44. Nyamweya, C.S., Natugonza, V., Taabu-Munyaho, A., Aura, C.M., Njiru, J.M., Ongore, C., Mangeni-Sande, R., Kashindye, B.B., Odoli, C.O., Ogari, Z., Kayanda, R., 2020. A century of drastic change: Human-induced changes of Lake Victoria fisheries and ecology. Fish. Res. 230, 105564. https://doi.org/10.1016/j.fishres.2020.105564.

45. Pietroiusti, Rosa and Vanderkelen, Inne and Otto, Friederike E. L and Barnes, Clair and Temple, Lucy and Akurut, Mary and Bally, Philippe and van Lipzig, Nicole P. M and Thiery, Wim. (2024). Possible role of anthropogenic climate change in the record-breaking 2020 Lake Victoria levels and floods. Earth System Dynamics. 15. 225-264. https://doi.org/10.5194/esd-15-225-2024.

46. Hamilton, S. (2018). Earth and Environmental Sciences". *Lake Victoria Statistics from this Dataverse* (Data Set). Harvard Dataverse. doi:10.7910/DVN/FVJJ4A

47. Sitoki, L., Gichuki, J., Ezekiel, C., Wanda, F., Mkumbo, O.C., Marshall, B.E., 2010. The

environment of Lake Victoria (East Africa): current status and historical changes. Int. Rev. Hydrobiol. 95, 209–223. https://doi.org/10.1002/iroh.201011226.

48. National Council for Population and Development , NCPD. (2021). State of Kenya population report 2021. National Government of Kenya, Ministry of Planning, Nairobi

49. Kenya National Bureau of Statistics, KNBS. (2023). Compendium of Environment Statistics 2023. National Government of Kenya, Ministry of State for Planning and National Sevelopment, Nairobi. P 184.

50. Kenya Meteorological Department, KMD (2023). National Framework for Climate Services Kenya – 2023. National Government of Kenya, Ministry of Environment, Climat Change and Forestry, Nairobi

51. USGS (2025). Landsat-8 L1. EarthExplorer Data archive - United States Geological Survey. US Department of Interior, Reston, Virginia. https://earthexplorer.usgs.gov/

52. Schillaci, C., Braun, A., Kropáček, J. (2015). Terrain analysis and landform recognition. In Clarke, L., Nield, J. Eds. Geomorphological Techniques Online ed. British Society for Geomorphology. https://doi.org/10.13140/RG.2.1.3895.2802 pp1-18.

53. Różycka, M., Migoń, P., Michniewicz, A. (2016). Topographic wetness index and terrain ruggedness index in geomorphic characterization of landslide terrains, on examples from the Sudetes, SW

Zeitschrift für Geomorphologie, Supplementary Issues. 61(2): 61-80. https://doi.org/10.1127/zfg_suppl/2016/0328.

54. Ghunowa, K., MacVicar, B, Ashmore, P.. (2021). Stream power index for networks (SPIN) toolbox for decision support in urbanizing watersheds. Environmental Modelling and Software. 144(7):105185. https://doi.org/10.1016/j.envsoft.2021.105185.

55. Pérez-Cutillas, P., Benabdelouahab, S, Salhi, A. (2025). Mitigating erosion and enhancing sediment retention: a modeling approach to sustainable land management. *Earth Systems and Environ*. https://doi.org/10.1007/s41748-025-00660-9

56. Ali P.J.M, Faraj, R. H. (2014). Data Normalization and Standardization: A Technical Report. Machine Learning Technical Reports 1(1): 1-6.

57. Tilahun, A., Desta, H. (2023). Soil erosion modeling and sediment transport index analysis using USLE and GIS techniques in Ada'a watershed, Awash River Basin, Ethiopia. *Geosci. Lett.* 10, 57 https://doi.org/10.1186/s40562-023-00311-9

58. Moore, I., Burch, G. (1986). Sediment transport capacity of sheet and rill flow: application of unit stream power theory. Water Resources Research 22(8): 1350–1360

59. Moore, I. D., Wilson, J. P. (1992). Length-slope factors for the revised universal soil loss equation: simplified method of estimation. Journal of Soil and Water Conservation 47(5): 423–428.

60. R Core Team. 2024. R: A Language and environment for statistical computing R V4.4.1. Foundation for Statistical Computing, Vienna Austria

61. Bolker, B. M., M. Brooks, C. J. Clark, S. W. Geange, J. R. Poulsen, M. Steven, J. S. S.White. 2009. Generalized linear mixed models: a practical guide for ecology". *Trends in* Ecology and Evolution 24:127–135. https://dpoi.org/doi: 10.1016/j.tree.2008.10.008.

62. Kelejian, H. H., Robinson, D. P. (1998). A suggested test for spatial autocorrelation and/or heteroskedasticity and corresponding Monte Carlo results. Regional Science and Urban Economics 8(24): 389-417. https://doi.org/10.1016/S0166-0462(98)00007-6

63. Fox J, Weisberg S (2019) An R companion to applied regression, 3rd edn. Sage, Thousand Oaks CA *Hamilton, S. (12 November 2017). "Basin, Lake Victoria Watershed (inside), vector polygon, ~2015". Harvard Dataverse (Data Set). doi:10.7910/dvn/z5rmyd.*

64. Kleiber C, Zeileis A (2008) Applied econometrics with R. Springer, New York, USA

65. Brooks, M. E., K. Kristensen, K.J. van Benthem, A. Magnusson, C.W. Berg, A. Nielsen, H. J. Skaug, M. Mächler and B. M. Bolker. 2017 "glmmTMB balances speed and flexibility among packages for zero-inflated generalized linear mixed modeling". *The R Journal* (2): 378-400

66. R Core Team. 2025."Package DHARMa - Residual diagnostics for hierarchical (multi-level mixed) regression models. v 0.4.7". Foundation for Statistical Computing, Vienna Austria. http://florianhartig.github.io/DHARMa.

67. Symonds, M.R.E., Moussalli, A. (2011). A brief guide to model selection multimodel inference and model averaging in behavioural ecology using Akaike's information criterion. Behav. Ecol. Sociobiol. 65, 13–21.

68. Molder, B., Cockburn, J., Berg, A., Lindsay, J., Woodrow, K, (2015). Sediment-assisted nutrient transfer from a small, no-till, tile drained watershed in southwestern Ontario, Canada. Agriculture and Water Management 152: 31-40, https://doi.org/10.1016/j.agwat.2014.12.010

69. Rafiei Emam, A.; Kappas, M.; Linh, N.H.K.; Renchin, T. (2017). Hydrological modeling and runoff mitigation in an Ungauged Basin of Central Vietnam Using SWAT Model. *Hydrology*, *4*: 16. https://doi.org/10.3390/hydrology4010016

70. Oshunsanya, S. O., Li, Y., Yu, H. (2019). Vetiver grass hedgerows significantly reduce nitrogen and phosphorus losses from fertilized sloping lands. Science of the Total Environment 661: 86-94. 10.1016/j.scitotenv.2019.01.129.

71. Radoane, M., Rădoane, N., Dumitriu, D., Miclaus, C. (2008). Downstream variation in bed sediment size along the East Carpathian rivers: evidence of the role of sediment sources. Earth Surface Processes and Landforms. 33: 674 - 694. https://doi.org/10.1002/esp.1568.

72. Wohl, E., Bledsoe, B., Jacobson, R., Poff, N., Rathburn, S., Walters, D., Wilcox, A. (2015). The Natural Sediment Regime in Rivers: Broadening the Foundation for Ecosystem Management. BioScience. 65(4): 1-14.. https://doi.org/10.1093/biosci/biv002.

73. Lwanga, A., Tsingalia, M., Humphrey, A., Shilenje, Z W. (2022). Effects of sand-harvesting on river water quality and riparian soil physico-chemical properties. Open Journal of Ecology. 12: 570- 583. https://doi.org/10.4236/oje.2022.128032.

74. Borelli, P., Robinson, D.A., Fleischer, L.R. *et al.* (2017). An assessment of the global impact of 21st century land use change on soil erosion. Nature Communications 8: https://doi.org/10.1038/s41467-017-02142-7

75. Nilsson, L., Andersson, G. K. S., Birkhofer, K., Smith, H. G. (2017). Ignoring Ecosystem-Service Cascades Undermines Policy for Multifunctional Agricultural Landscapes. Frontiers in Ecology and Evolution 5:109. http://doi.org/10.3389/fevo.2017.00109

76. Menzies, N., Wang, P., McKenna, B., Lombi, E. (2019). Soil and the intensification of agriculture for global food security. Environment international. 132. 105078. https://doi.org/10.1016/j.envint.2019.105078. 132:105078

77. Rodríguez S. A. A., Muñoz-Rojas, J., Brígido, C., Prats, S. A, (2023). Impacts of agricultural intensification on soil erosion and sustainability of olive groves in Alentejo (Portugal). Landscape Ecology 38: 3479–3498.. https://doi.org/10.1007/s10980-023-01682-2

78. Diop, M., Chirinda, N., Beniaich, A., El Gharous, M., El Mejahed, K. (2022). Soil and Water Conservation in Africa: State of Play and Potential Role in Tackling Soil Degradation and Building Soil Health in Agricultural Lands. *Sustainability*, *14*(20), 13425. https://doi.org/10.3390/su142013425

79. Verma, N. S., Kishore, G., Gupta, D., Kuldeep, D. (2023). Tillage concept and its impact on crop production. In Verma et al, *Frontiers of Agronomy.* Elite Publishing House, New Dehli. Pp 80-93.

80. Gao, P. J., Abbas, H., Li, F. Q., Tang, G. R., Lv, J. Z., Zhou, X. B. (2024) Effect of planting methods and tillage practices on soil health and maize productivity. Frontiers in Plant Science. 15:1436011. https://doi.org/10.3389/fpls.2024.1436011

81. Adimassu, Z., Langan, S., Johnston, R., Mekuria, W., Amede, T. (2017). Impacts of soil and water conservation practices on crop yield, run-off, soil loss and nutrient loss in Ethiopia: review and synthesis. Environmental Management 59(1): pp. 87-101. https://doi.org/10.1007/s00267-016-0776-1

82. Iseyemi, O. O., Farris, J. L., Moore, M. T., Choi, S. E. (2016). Nutrient mitigation efficiency in agricultural drainage ditches: an influence of landscape management. Bulletin of

Environmental Contamination and Toxicology 96 (6): 750-756, https://doi.org/10.1007/s00128-016-1783-x

83. Goldman, M. A., Needelman, B. A. (2015). Wetland restoration and creation for nitrogen removal: Challenges to developing a watershed-scale approach in the Chesapeake Bay coastal plain. In D.L. Sparks (Ed.), *Advances in Agronomy*, Vol. 132, Elsevier Academic Press Inc, San Diego. pp. 1-38

84. Gassman, P. W., Osei, E., Saleh, A., Rodecap, J., Norvell, S., Williams, J. (2006). Alternative practices for sediment and nutrient loss control on livestock farms in Northeast Iowa. Agriculture, Ecosystems and Environment 117(2–3): 135-144. https://doi.org/10.1016/j.agee.2006.03.030

85. Fennessy, S., Craft, C. (2011). Agricultural conservation practices increase wetland ecosystem services in the Glaciated Interior Plains. Ecological Applications 21(3 SUPPL.): 49-64. https://doi.org/10.1890/09-0269.1

86. Irwin, N. B., Irwin, E. G., Martin, J. F., Aracena, P. (2018). Constructed wetlands for water quality improvements: benefit transfer analysis from Ohio. Journal of Environmental Management 206: 1063-1071. https://doi.org/10.1016/j.jenvman.2017.10.050

87. Tesha, D., Mauya, E., Madundo, S., Emily, C. (2023). Role of topography, soil and climate on forest species composition and diversity in the West Usambara Montane Forests of Tanzania. Folia Oecologica. 50: 105-118. https://doi.org/10.2478/foecol-2023-0010.

88. Antolini, F., Tate, E., Dalzell, B., Young, N., Johnson, K., Hawthorne, P. (2019). Flood risk reduction from agricultural best management practices. Journal of the American Water Resources Association. 56(1):. https://doi.org/10.1111/1752-1688.12812.

89. Quian, X., Zhao, L., Fang, Q., Fan, C., Zi, R., Fang, F. (2023). Rill formation and evolution caused by upslope inflow and sediment deposition on freshly tilled loose surfaces. Soil and Tillage Research. 235(264):105868. 10.1016/j.still.2023.105868.

90. Schultz, R. C., Isenhart, T. M., Simpkins, W. W., Colletti, J. P. (2004). Riparian forest buffers in agroecosystems - lessons learned from the Bear Creek watershed, Central Iowa, USA. Agroforestry Systems 61–62(1–3): 35-50.

91. Rajaonarimalala, R., Korol, Y., Andrianarimisa, A., Dröge, S., Fulgence, T., Grass, I., Kreft, H., Osen, K., Rakotomalala, Anjaharinony A., Rakouth, B., Ranarijaona, H., Schwab, D., Soazafy, M., Tscharntke, T., Wurz, A., Martin, D. (2024). Complex stands in forested tropical landscapes harbor more endemic biodiversity and ecosystem functions. Global Ecology and Conservation. 54. e03154.. 54(3):e03154. https://doi.org/10.1016/j.gecco.2024.e03154

92. Rustomji, P., Prosser, I. (2001). Spatial patterns of sediment delivery to valley floors: Sensitivity to sediment transport capacity and hillslope hydrology relations. Hydrological Processes. 15:. 1003 - 1018. https://doi.org/10.1002/hyp.231.

93. Wang, N., Luo, J., Zheng, Z., Li, T., He, S., Zhao, Y., Wang, Y., Zhang, X., Huang, H., Yu, H., Ye, D., Liu, T. (2023). Upslope inflow-driven variations in microtopography and size fractions effect on rill erosion in purple soil hillslopes. Journal of Hydrology. 622(3): 129759. https://doi.org/10.1016/j.jhydrol.2023.129759.

94. Quian, J., Ji, C., Yang, J., Zhao, H., Wang, Y., Fu, L., Qiang, L. (2024). The advantage of afforestation using native tree species to enhance soil quality in degraded forest ecosystems. Scientific Reports. 14(1): 1-10.. https://doi.org/10.1038/s41598-024-71162-3.

95. Heckmann, T., Schwanghart, W. (2013(. Geomorphic coupling and sediment connectivity in an alpine catchment - Exploring sediment cascades using graph theory. Geomorphology 182: 89-103

96. Jantiene E. M., Baartman,D., Masselink, R., Keesstra, S. D., Temme, A. J. (2013). Linking landscape morphological complexity and sediment connectivity. Earth Surface Processes and Landforms 38: 1457–1471.

97. Najafi, S., Dragovich, D., Heckmann, T., Sadeghi, S. H. (2020). Sediment connectivity concepts and approaches. Catena. 196(12): 104880. https://doi,org/10.1016/j.catena.2020.104880.

98. Kogo, B. K., Kumar, L., Koech, R. (2019). Forest cover dynamics and underlying driving forces affecting ecosystem services in western Kenya. Remote Sensing Applications: Society and Environment 14: 75-83. https://doi.org/10.1016/j.rsase.2019.02.007

99. Opiyo, S., Opinde, G., Letema, S. (2022). Dynamics and drivers of land use and land cover changes in Migori River watershed, western Kenya region. Watershed Ecology and the Environment. 4(11): 219-232. https://doi.org/10.1016/j.wsee.2022.11.008.

100. Qiu, Z. (2003). A VSA-based strategy for placing conservation buffers in agricultural watersheds Environtal Management 32(3): 299-311. https://doi.org/10.1007/s00267-003-2910

101. Kipsat, M., Mose, P., Osewe, D. (2021). A probit analysis of factors affecting decisions to continue maintaining soil conservation structures after adoption of the technology in Kericho County, Kenya. Russian Journal of Agricultural and Socio-Economic Sciences. 119: 147-153. https://doi.org/10.18551/rjoas.2021-11.17.

102. Rotich, B., Ojwang', D. (20212). Trends and drivers of forest cover change in the Cherangany hills forest ecosystem, western Kenya. Global Ecology and Conservation 30:, e01755. https://doi.org/10.1016/j.gecco.2021.e01755

103. Zhang, G. H., Wang, L. L., Tang, K. M., Luo, R. T., and Zhang, X. C. (2011). Effects of sediment size on transport capacity of overland flow on steep slopes. *Hydrological Sciences Journal*, *56*(7), 1289–1299. https://doi.org/10.1080/02626667.2011.609172

104. Emirhüseyinoğlu, G., Ryan, S. M. (2020). Land use optimization for nutrient reduction under stochastic precipitation rates Environtal Modeling and Sorftware 123 https://doi.org/10.1016/j.envsoft.2019.104527

105. Tournebize, J., Chaumont, C., Mander, Ü. (2017). Implications for constructed wetlands to mitigate nitrate and pesticide pollution in agricultural drained watersheds. Ecological Engineering 103: 415-425. https://doi.org/10.1016/j.ecoleng.2016.02.014

106. Herath, P., Croke, B., Prinsley, R., Vaze, J., Pollino, C. (2025). A systematic review of forest cover for catchment-scale flood mitigation: a nature-based solution. Journal of Flood Risk Management. 18(3): 1 -20. https://doi.irg/10.1111/jfr3.70125.

107. Greenberg, J. A., Santos, M. J., Dobrowski, S. Z, Vanderbilt, V.C., Ustin, S .L. (2015). Quantifying environmental limiting factors on tree cover using geospatial data. PLoS ONE 10(2): e0114648. https://doi.org/10.1371/journal.pone.0114648

108. Evans, J. (2007). Sediment impacts of the 1994 failure of IVEX Dam (Chagrin River, NE O hio): A test of channel evolution models. Journal of Great Lakes Research 33: 90-102. https://doi.org/10.3394/0380-1330(2007)33[90:SIOTFO]2.0.CO;2.

109. Adamo, N., Al-Ansari, N., Sissakian, V., Laue, J., Knutsson, S. (2020). Dam safety: sediments and debris problems. Journal of Earth Sciences and Geotechnical Engineering. 11: 27-63. https://doi.org10.47260/jesge/1112.

Figure Legends

Fig 1. Map of study area indicating the focal area in Kenyan side of Lake Victoria encompassing the counties which form the catchment of the lake.

Fig 2. Map of the selected study are indicating: The mosaicked separate panels of the satellite imagery in A) Pseudo-color and B) Grey-scale; C) Indicating the constituent counties on hillshade; D) Showing the entire stream channel network; E) Showing major rivers entering Lake Victoria; and F) Showing the all major rivers network and counties in re-projected form.

Fig 3. Representation of locations and sizes of the six identified major catchment zones and their associated channel networks potentially contributing to erosion and sediment transport into Lake Victoria

Fig 4. County rankings for A) net erosion indicator (NEI); B) sediment transport indices (STI); and C) overall erosivity potential (ErPot).

Fig 5. Regression plots of the predicted trends net erosion indicator response to A)

Terrain ruggedness, B) total cropland size and C) Total area covered by wetland habitat.

Fig 6. Regression plots of the predicted trends sediment transport index response to A)

Slope size; B) Elevation; C) Area covered by forest; and C) Total area covered by wetland habitat

Fig 7. Regression plots of the predicted trends overall erosion potential (ErPot) response to A)

A) Area covered by cropland; and B) Total area covered by forest.

Supporting Information captions

S1 Fig. Results of model fit diagnostic tests, indicating observed Quantile-Quantile and DHARMa residual plots for data distribution, (KS), dispersion and outliers against

expected scenarios, for the three response variables A) The fit for the net erosion indicator - NEI ; B) The fit for sediment transport index – STI; and C) overall erosivity potential – ErPot.

S2 Fig. Variance Inflation Factor (VIF) test results for variables retained after selecting best models

S1 File: List of the best models selected with the respective retained variables for regression modelling.(NOTE: log=logarithm to base 10; tri=terrain ruggedness index; slope=mean slope angle; elev= mean elevation; forest=percent forest cover; crop=percent crop cover; wetlandcover; percent cover of wetland habitat; urbancover-total area covered by urban infrastructure;)

1. Net erosion indicator (NEI)

modNEI<-glmm(logeros~meantri+logslope+logelev+logforest+logcrop+logwetlandcover+logUrbancover(1|countysize), family = beta, link – logit, data=mine)

2. Sediment transport index (STI)

modSEDIMENT<-glmm(STI~+meantri+logslope+logelev+logforest+logcrop+logwetlandcover+logurbancover+(1|countysize), family = beta, link – logit, data=sed)

3. Erosivity potential (ErPoT) = NEI+STI

modErPot<-glmm(logerosandsti~logforest+logcrop+urbancover, family = beta, link – logit, data=nickdata)

Figure 1

Click here to access/download;Figure;Fig 1.tif

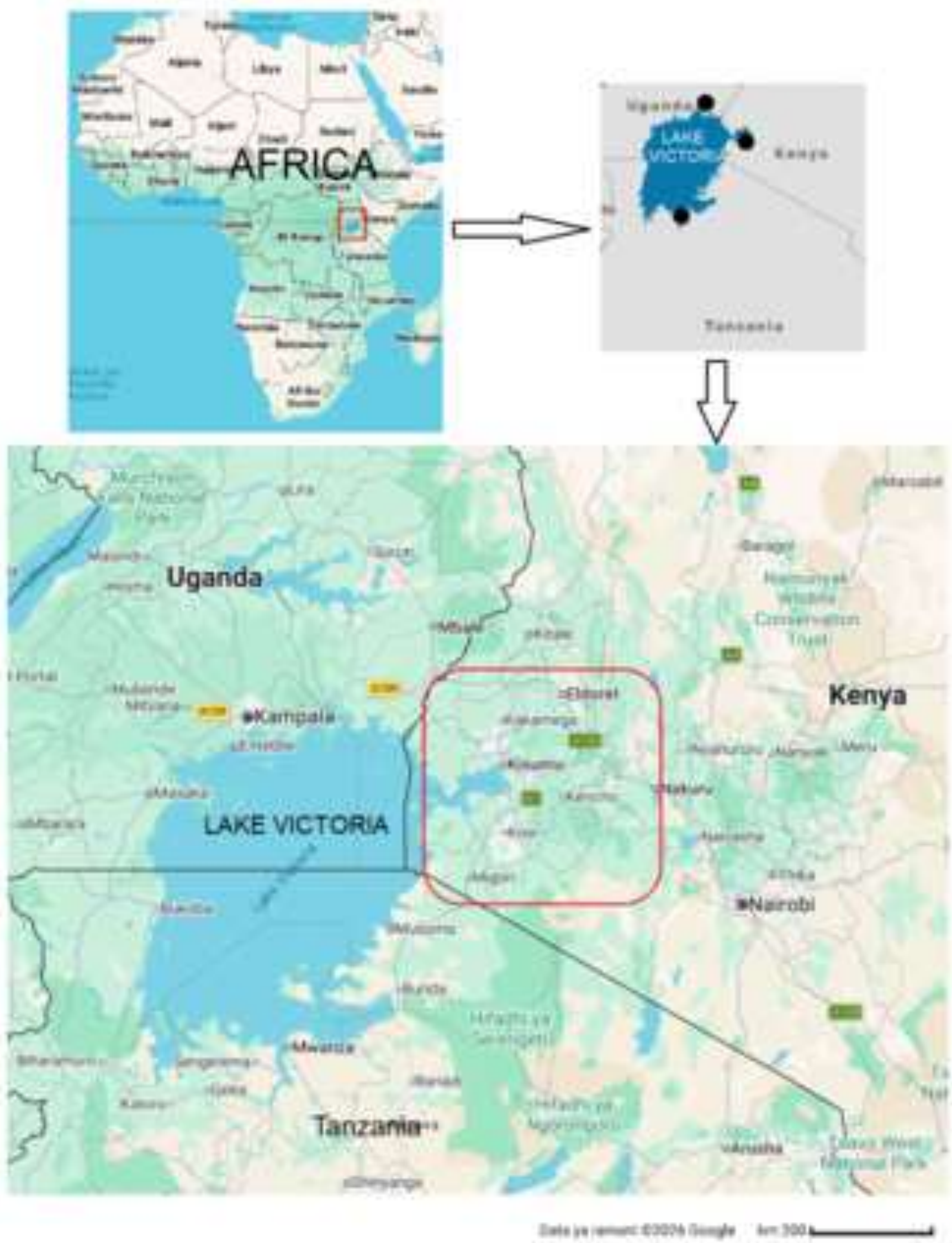

Figure 2

Click here to access/download;Figure;Fig 2.tif

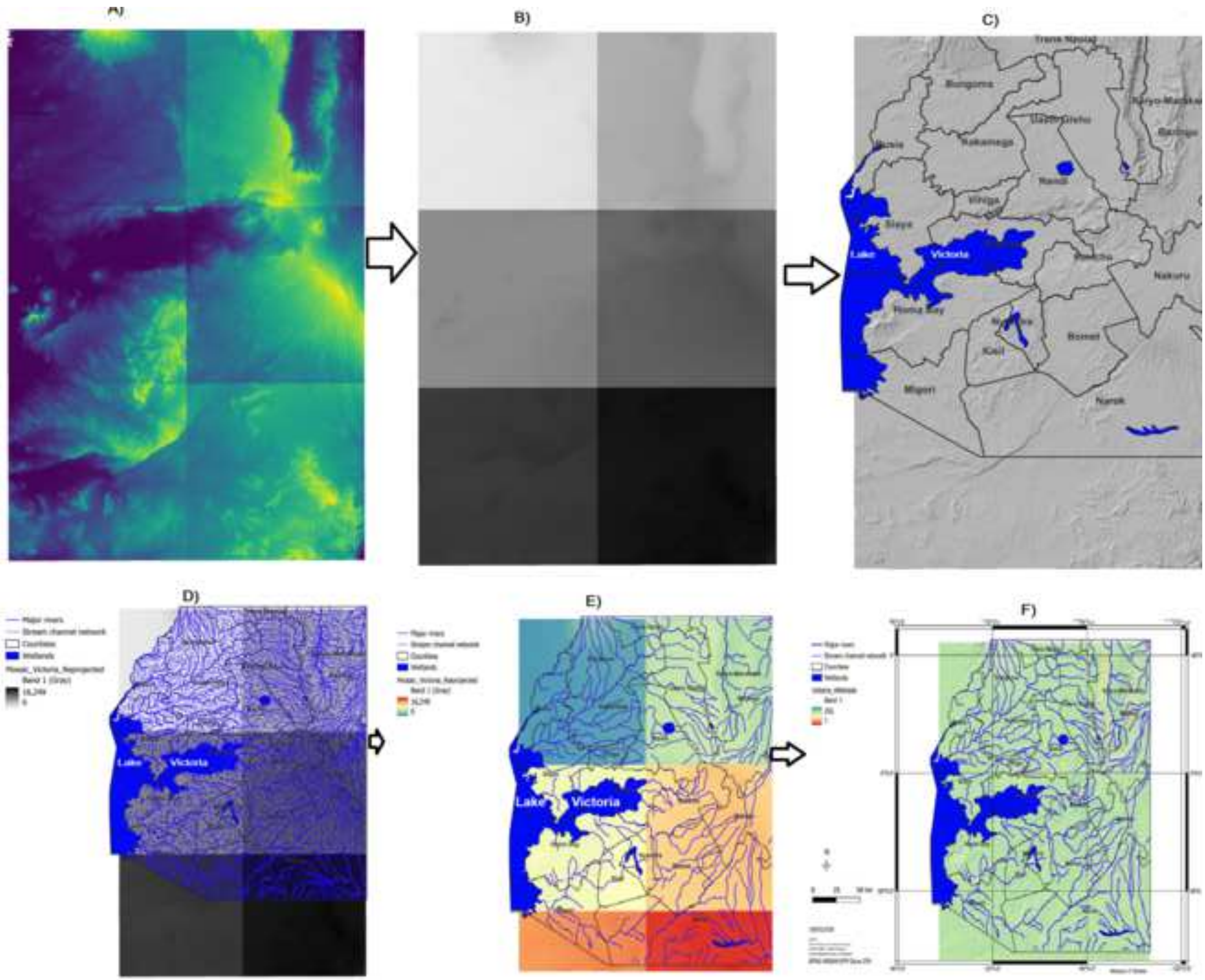

Figure 3

Click here to access/download;Figure;Fig 3.tif

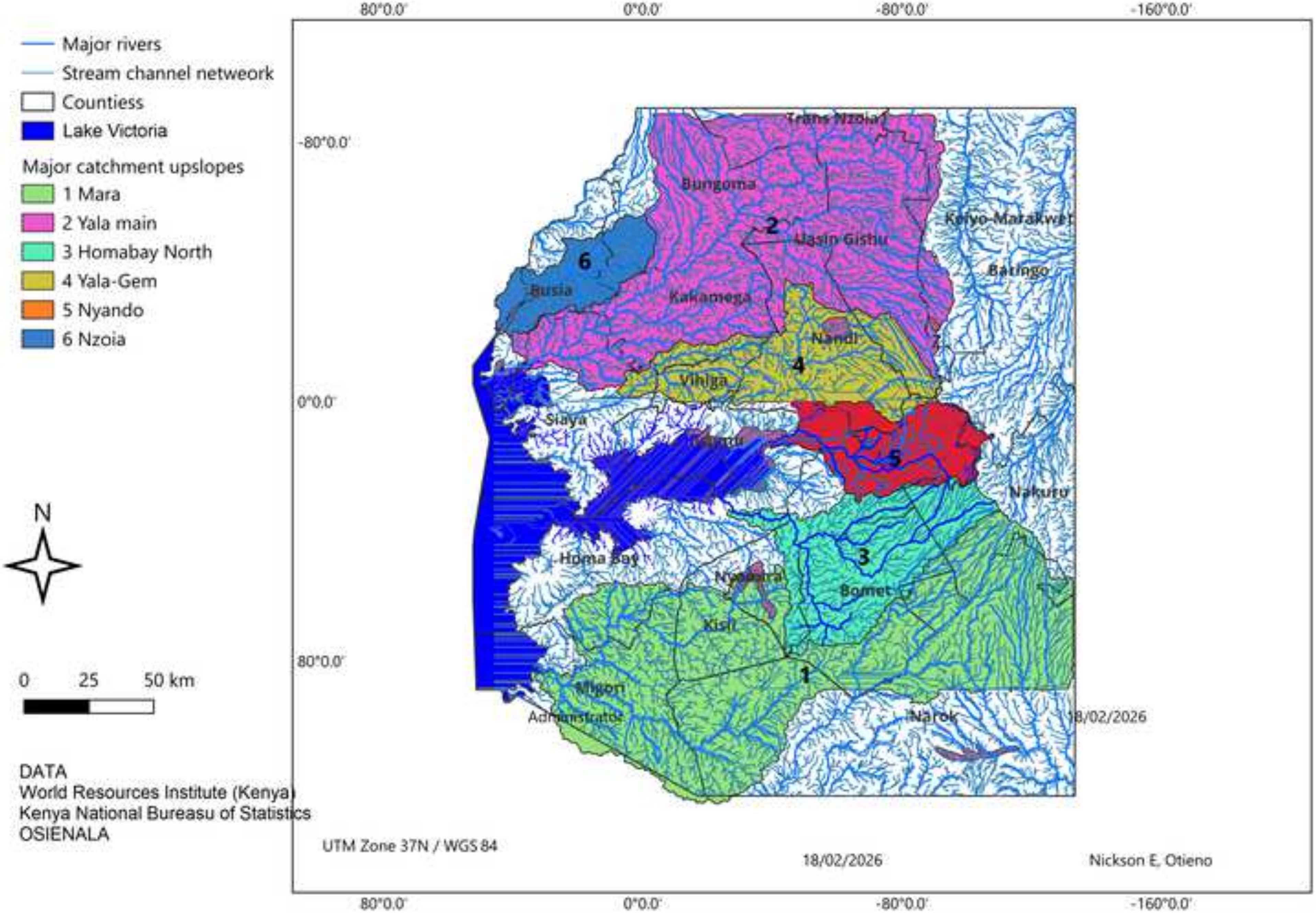

Figure 4

Click here to access/download;Figure;Fig 4.tif

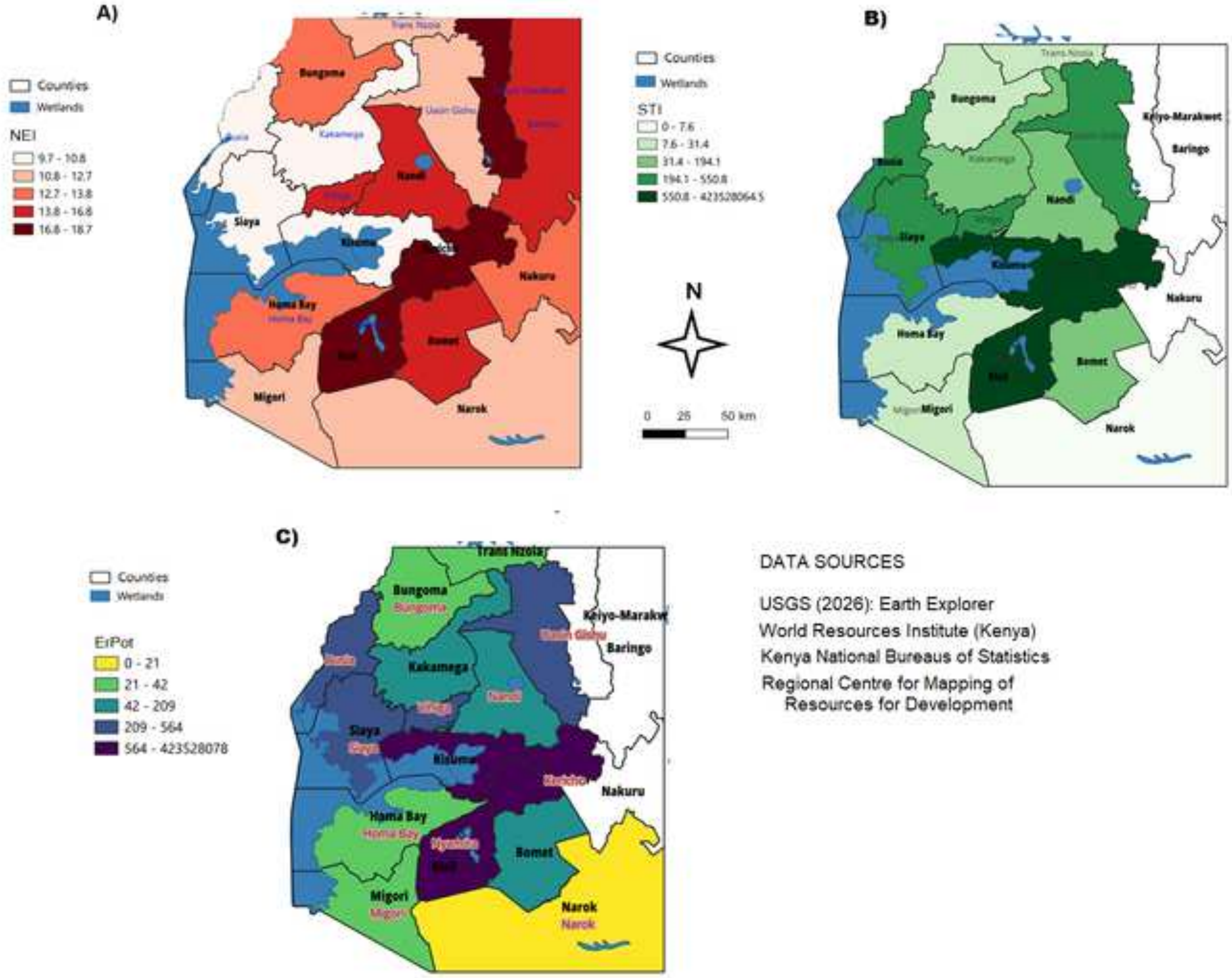

Figure 5

Click here to access/download;Figure;Fig 5.tif

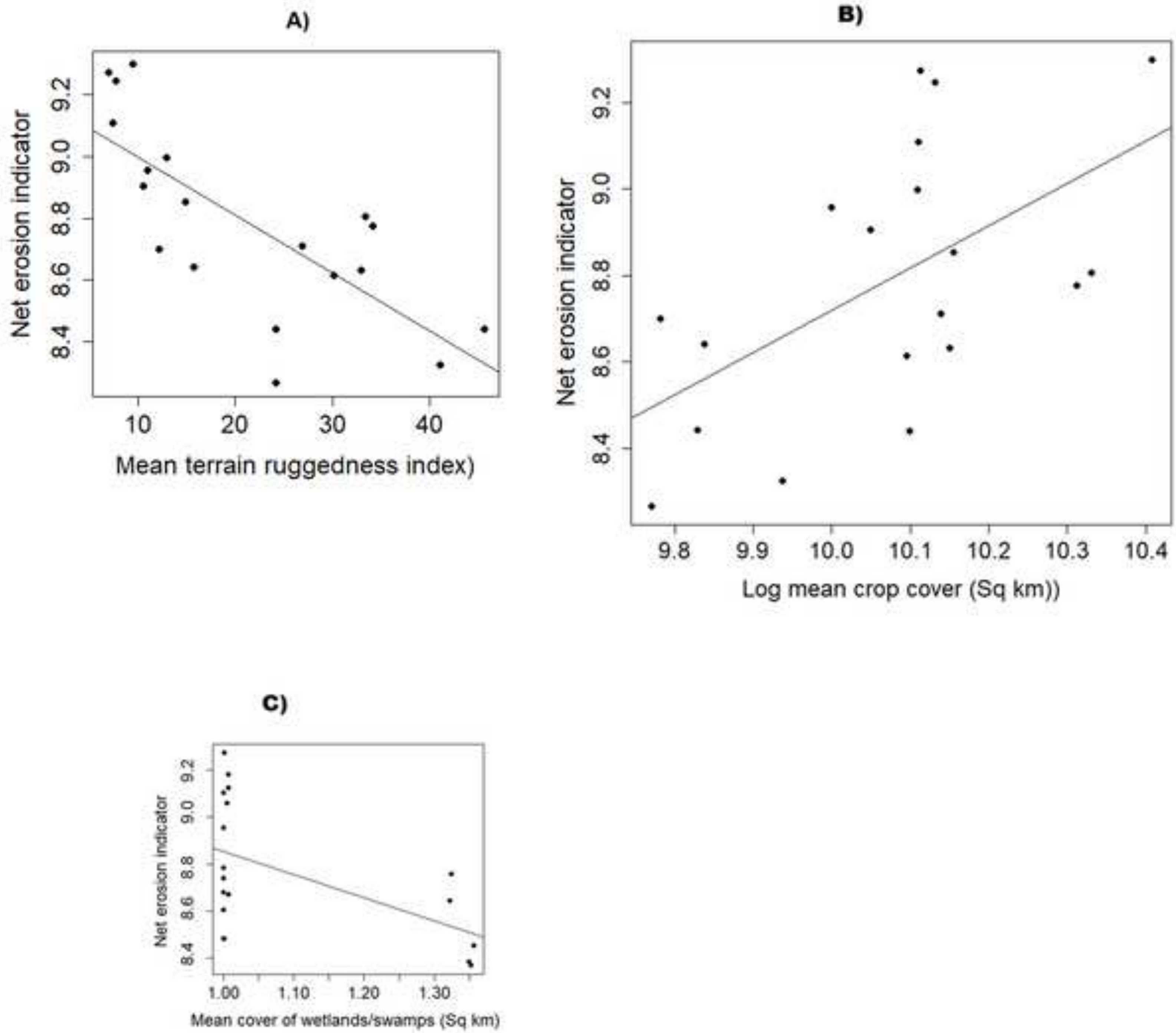

Figure 6

Click here to access/download;Figure;Fig 6.tif

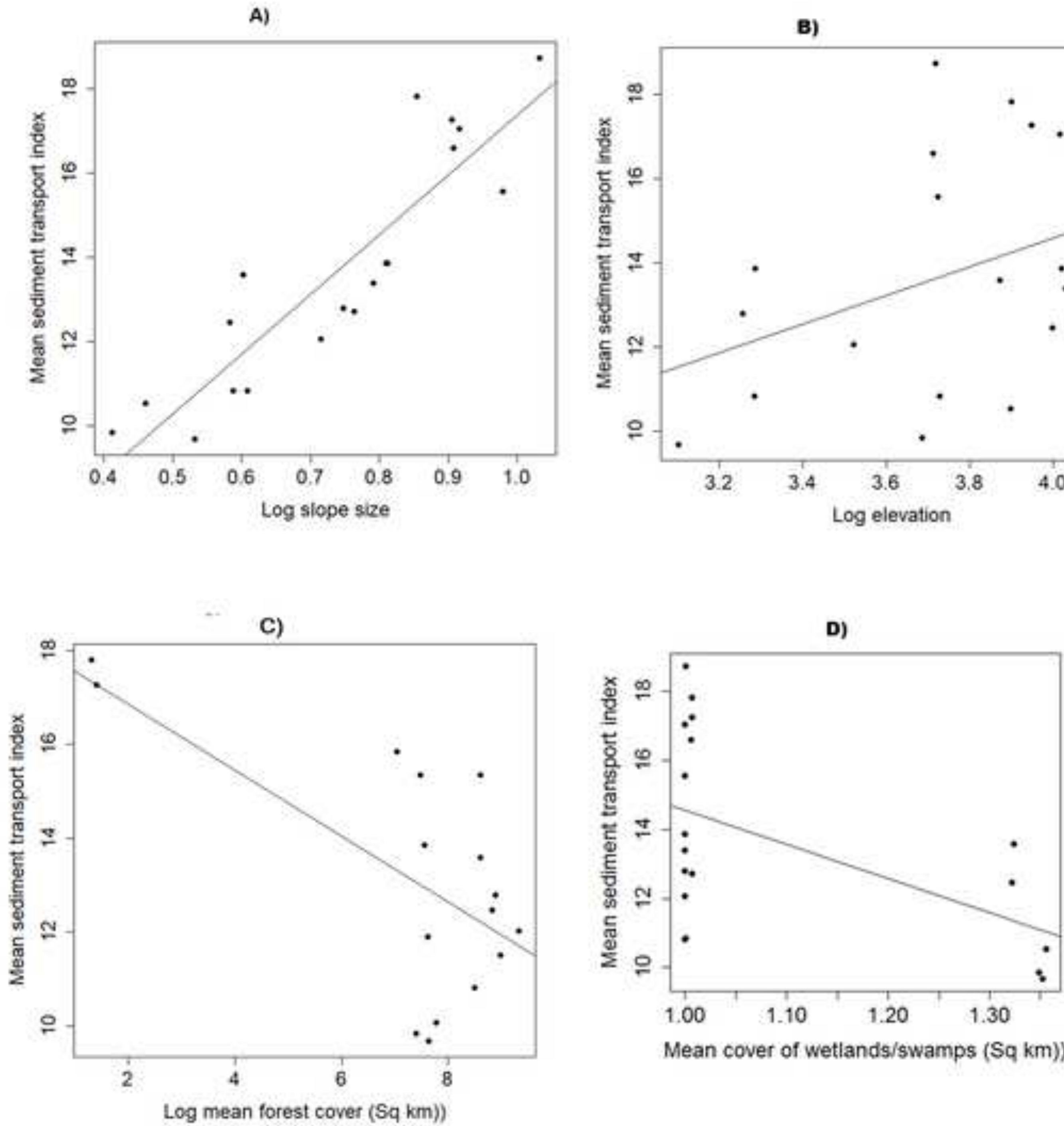

Figure 7

Click here to access/download;Figure;Fig 7.tif

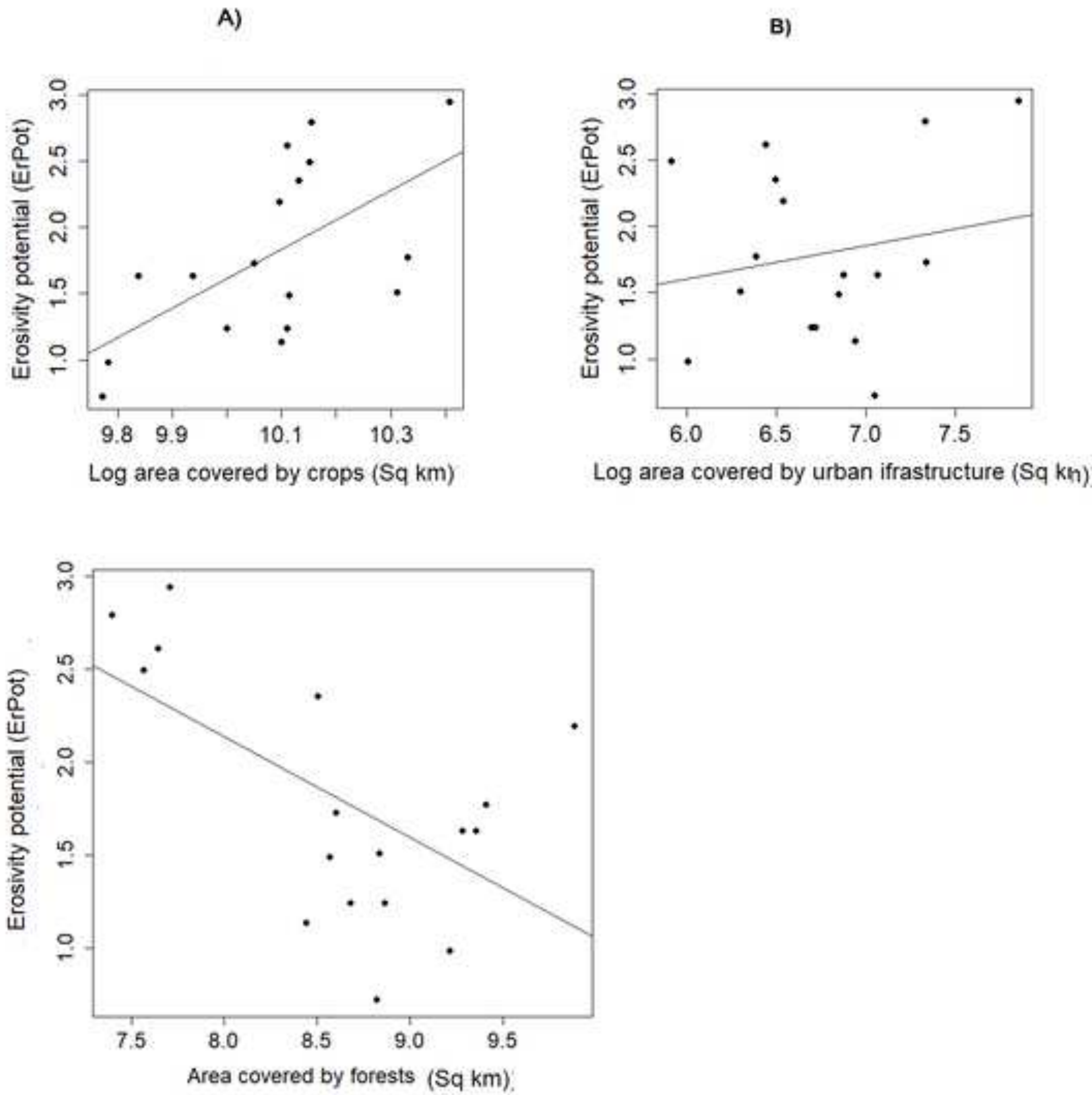

S1 Figure

Click here to access/download
Supporting Information
S1 Fig.tif

S2 Figure

Click here to access/download
Supporting Information
S2 Fig.tif

S1 File

Click here to access/download
Supporting Information
S1 File.pdf

PONE-D-26-12960: Using linear modeling and remote sensing to identify priority spatial and parameter-based mitigation measures against erosion and sediment movement within Lake Victoria watershed in Kenya, by Nickson E Otieno, PhD

(Originally PONE-D-26-10754: Remote sensing for prioritizing spatial and parameter-based mitigation measures against erosion and sediment movement within the Lake Victoria watershed in Kenya)

Dear EDITOR,

Thank you the opportunity to revise/edit and resubmit my article.

The Academic Editor made a unilateral decision on this submission without sending it out to any review examination. Therefore, I had no reviewer comments to work on for revision.

The concerns raised were very few, very brief and very overally generalized, with no in-depth exposition of any specific issues that rendered the decision for a decision of immediate rejection.

In originally submitting the paper, I had carefully adhered to all submission guidelines for PLOS ONE, including adherence to inclusion of all required sections for a research paper, scope, organization, formatting and submission of supporting materials. I also duly adhered to the scientific rigor in terms of literature review, identification I of research gaps, justification or significance of the study, methodological protocol, analysis approached and steps, presentation of results and discussing the results to place it them within the context of our stated aims and objectives, as well as that of previous studies. Finally, our conclusion entailed summarizing our findings and outlining the extent to which of our hypotheses were realized or not. I even ended by stating limitations of our study and recommending possible future research directions that could potentially minimize those limitations. Therefore, I believe our article already met the required scientific rigor expected, and also complied fully with the required standards and article guidelines of PLOS ONE.

In the revised version of the paper, I have provided an even more improved version in which I have addressed point by point, each of the generalized issues highlighted by the Academic EDITOR, pointing out the sections and lines in the manuscript where I have made the specific changes recommended (also highlighted in color). I have also submitted a track-changed version of the revised paper as instructed by the Editorial Office.

Additional improvements I have made include

A more careful and re-writing of the Abstract to improve appreciation of the study's focus and key findings as well as highlight significance pf the study and applicability of the results obtained.

A modified main title

A slightly modified short title

Modified key words

I have also ensured that the equations and formulae are formatted using the recommended equation tools, and presented in the standard style required by PLOS ONE

I have also enhanced the quality of the figures to comply with the journal's publication standards.

Finally, I have rechecked the whole article for grammatical mistakes of spelling to further improve readability.

Below is a detailed account of how I have addressed the generalized issues raised by the Academic EDITOR; The Author responses are highlighted in green

Additional ACADEMIC EDITOR Comments:

Dear authors,

After a careful review of your manuscript, I find that

ACADEMIC EDITOR COMMENT: the novelty of the work is not clearly articulated.

AUTHOR'S RESPONSE: The novelty of the study is already clearly articulated in Line 118 to Line 122

ACADEMIC EDITOR COMMENT Furthermore, the formula presented does not follow standard formatting conventions,

AUTHOR'S RESPONSE: The formula and equations presented are now formatted using the Equation Tools as required by PLOS ONE

ACADEMIC EDITOR COMMENT the visualization of the experimental results does not meet the expected academic standards.

AUTHOR'S RESPONSE: The ACADEMIC EDITOR did not any specific way in which the academic standards of the visualized figures were not met. Nevertheless, I have enhanced the quality of the figures, improving sizes and resolutions to improve readability and visibility, as well as applied the PLOS ONE figure formatting tool (NAAS) to bring them up to the standards expected by the journal. Font sizes for Tables and table captions have also been increased to comply with the journal's requirements

ACADEMIC EDITOR COMMENT As it currently stands, I recommend that the quality of the submission be improved. This should include a more thorough discussion,

AUTHOR'S RESPONSE: The ACADEMIC EDITOR did not specify any aspects of the Discussion that were not thorough enough. Nonetheless I have now expanded on the points presented in the section, adding more references to previous research as well as linked them to relevant citations

ACADEMIC EDITOR COMMENT a professionally grounded explanation of the study's rationale,

AUTHOR'S RESPONSE: A well-grounded rationale of the study was already clearly articulated in the article Introduction (Line 122 to Line 126), but also in the final sentence of the Abstract, as well as the Conclusion (Line 550 – Line 555

ACADEMIC EDITOR COMMENT and a clearer presentation of the experimental results.

AUTHOR'S RESPONSE: The ACADEMIC EDITOR did not mention any way in which the results were not clearly presented, but I have clearly divided the \results into various subsections corresponding the objectives, and also to correspond to each of the methodological-protocol and analysis steps to facilitate easier flow and readability. I have also provided more enhanced visual results (figures).

ACADEMIC EDITOR COMMENT It is also essential to explicitly highlight the novelty of the manuscript,

AUTHOR'S RESPONSE: The novelty of the study is already clearly articulated in Line 118 to Line 122

ACADEMIC EDITOR COMMENT: clearly state the motivation for conducting the study and identify the research gaps

AUTHOR'S RESPONSE: The motivation for the study and specification of research gaps were already clearly presented in the Introduction (Line 116 to Line 119.

ACADEMIC EDITOR COMMENT questions the work aims to address.

AUTHOR'S RESPONSE: The questions the work aimed to address (the general as and specific objectives as well as what I expected to find (hypotheses) were all already clearly articulated towards the end of introduction (Line 108 to Line 123).
