# Supplementary material for: Using GIS, remote-sensing and linear modeling to prioritize spatially explicit action against erosion and sediment transport within Lake Victoria watershed in Kenya
Source: PLoS One. 2026 Oct 1;21(10):e0359542. doi: 10.1371/journal.pone.0359542 (PMC13630186; doi:10.1371/journal.pone.0359542)
Supplement: Supplementary file 4 [file pone.0359542.s006.docx]

**PONE-D-26-12960: Using linear modeling and remote sensing to identify priority spatial and parameter-based mitigation measures against erosion and sediment movement within Lake Victoria watershed in Kenya,** by Nickson E Otieno, PhD

*(Originally PONE-D-26-10754: Remote sensing for prioritizing spatial and parameter-based mitigation measures against erosion and sediment movement within the Lake Victoria watershed in Kenya)*

Dear EDITOR,

Thank you the opportunity to revise/edit and resubmit my article.

The Academic Editor made a unilateral decision on this submission without sending it out to any review examination. Therefore, I had no reviewer comments to work on for revision.

The concerns raised were very few, very brief and very overally generalized, with no in-depth exposition of any specific issues that rendered the decision for a decision of immediate rejection.

In originally submitting the paper, I had carefully adhered to all submission guidelines for PLOS ONE, including adherence to inclusion of all required sections for a research paper, scope, organization, formatting and submission of supporting materials. I also duly adhered to the scientific rigor in terms of literature review, identification I of research gaps, justification or significance of the study, methodological protocol, analysis approached and steps, presentation of results and discussing the results to place it them within the context of our stated aims and objectives, as well as that of previous studies. Finally, our conclusion entailed summarizing our findings and outlining the extent to which of our hypotheses were realized or not. I even ended by stating limitations of our study and recommending possible future research directions that could potentially minimize those limitations. Therefore, I believe our article already met the required scientific rigor expected, and also complied fully with the required standards and article guidelines of PLOS ONE.

In the revised version of the paper, I have provided an even more improved version in which I have addressed point by point, each of the generalized issues highlighted by the Academic EDITOR, pointing out the sections and lines in the manuscript where I have made the specific changes recommended (also highlighted in color). I have also submitted a track-changed version of the revised paper as instructed by the Editorial Office.

Additional improvements I have made include

A more careful and re-writing of the Abstract to improve appreciation of the study’s focus and key findings as well as highlight significance pf the study and applicability of the results obtained.

A modified main title

A slightly modified short title

Modified key words

I have also ensured that the equations and formulae are formatted using the recommended equation tools, and presented in the standard style required by PLOS ONE

I have also enhanced the quality of the figures to comply with the journal’s publication standards.

Finally, I have rechecked the whole article for grammatical mistakes of spelling to further improve readability.

Below is a detailed account of how I have addressed the generalized issues raised by the Academic EDITOR; The Author responses are highlighted in green

Additional ACADEMIC EDITOR Comments:

Dear authors,

After a careful review of your manuscript, I find that

ACADEMIC EDITOR COMMENT: the novelty of the work is not clearly articulated.

**AUTHOR’S RESPONSE:** The novelty of the study is already clearly articulated in Line 118 to Line 122

ACADEMIC EDITOR COMMENT Furthermore, the formula presented does not follow standard formatting conventions,

**AUTHOR’S RESPONSE:** The formula and equations presented are now formatted using the Equation Tools as required by PLOS ONE

ACADEMIC EDITOR COMMENT the visualization of the experimental results does not meet the expected academic standards.

**AUTHOR’S RESPONSE:** The ACADEMIC EDITOR did not any specific way in which the academic standards of the visualized figures were not met. Nevertheless, I have enhanced the quality of the figures, improving sizes and resolutions to improve readability and visibility, as well as applied the PLOS ONE figure formatting tool (NAAS) to bring them up to the standards expected by the journal. Font sizes for Tables and table captions have also been increased to comply with the journal’s requirements

ACADEMIC EDITOR COMMENT As it currently stands, I recommend that the quality of the submission be improved. This should include a more thorough discussion,

**AUTHOR’S RESPONSE:** The ACADEMIC EDITOR did not specify any aspects of the Discussion that were not thorough enough. Nonetheless I have now expanded on the points presented in the section, adding more references to previous research as well as linked them to relevant citations

ACADEMIC EDITOR COMMENT a professionally grounded explanation of the study's rationale,

**AUTHOR’S RESPONSE:** A well-grounded rationale of the study was already clearly articulated in the article Introduction (Line 122 to Line 126), but also in the final sentence of the Abstract, as well as the Conclusion (Line 550 – Line 555

ACADEMIC EDITOR COMMENT and a clearer presentation of the experimental results.

**AUTHOR’S RESPONSE:** The ACADEMIC EDITOR did not mention any way in which the results were not clearly presented, but I have clearly divided the \results into various subsections corresponding the objectives, and also to correspond to each of the methodological-protocol and analysis steps to facilitate easier flow and readability. I have also provided more enhanced visual results (figures).

ACADEMIC EDITOR COMMENT It is also essential to explicitly highlight the novelty of the manuscript,

**AUTHOR’S RESPONSE:** The novelty of the study is already clearly articulated in Line 118 to Line 122

ACADEMIC EDITOR COMMENT: clearly state the motivation for conducting the study and identify the research gaps

**AUTHOR’S RESPONSE:** The motivation for the study and specification of research gaps were already clearly presented in the Introduction (Line 116 to Line 119.

ACADEMIC EDITOR COMMENT questions the work aims to address.

**AUTHOR’S RESPONSE:** The questions the work aimed to address (the general as and specific objectives as well as what I expected to find (hypotheses) were all already clearly articulated towards the end of introduction (Line 108 to Line 123).
