# Supplementary material for: Using GIS, remote-sensing and linear modeling to prioritize spatially explicit action against erosion and sediment transport within Lake Victoria watershed in Kenya
Source: PLoS One. 2026 Oct 1;21(10):e0359542. doi: 10.1371/journal.pone.0359542 (PMC13630186; doi:10.1371/journal.pone.0359542)
Supplement: Supplementary file 5 [file pone.0359542.s007.docx]

**PONE-D-26-12960: Using GIS, remote-sensing and linear modeling to prioritize spatially explicit action for mitigating erosion and sediment transport within Lake Victoria watershed in Kenya**

by Nickson E Otieno, PhD

Dear EDITOR,

Thank you the opportunity to revise/edit and resubmit my article.

In the revised version of the paper, I have provided an considerably improved version in which I have addressed point by point, each of the generalized issues highlighted by the reviewers, pointing out the sections and lines in the manuscript where I have made the specific changes recommended (also highlighted in color). I have also submitted a track-changed version of the revised paper as instructed by the Editorial Office.

Additional improvements I have made include:

Basing all analyses and evaluation of the erosion and sediment processes on the spatial scale or sub-watershed rather than at administrative county levels as before

A more careful and re-writing of the Abstract to improve appreciation of the study’s focus and key findings as well as highlight significance pf the study and applicability of the results obtained.

A modified main title

A slightly modified short title

I have also enhanced the quality of the figures to comply with the journal’s publication standards.

Finally, I have rechecked the whole article for grammatical mistakes of spelling to further improve readability.

Below is a detailed account of how I have addressed the generalized issues raised by the Academic EDITOR; The Author responses are highlighted in green

**Reviewers' comments:**

**Reviewer #1:**

 As a reviewer for the PLOS ONE journal, here is an in-depth and critical review report of the manuscript titled Using linear modeling and remote sensing to identify priority spatial and parameterbased mitigation measures against erosion and sediment movement within Lake
Victoria watershed in Kenya.
This study presents original research integrating remote sensing data (Landsat 8) and statistical modeling (GLMM) to assess the Net Erosion Indicator (NEI), Sediment Transport Index (STI), and Overall Erosion Potential (ErPot) in 19 districts of the Lake Victoria watershed, Kenya. The data and analysis presented are the result of new calculations by the authors.

1. Evaluation of Novelty for Journal Standard
Recommendation Regarding Novelty: Moderate (Does Not Fully Meet Standard Q1 Without Significant Revision).
• Strength of Novelty: The authors claim that the novelty of this study lies in the comprehensive evaluation of the Sediment Transport Index (STI) within the context of large-scale regional (district-level) spatial modeling, rather than simply point mapping, and its integration with the Net Erosion Indicator (NEI) in a large tropical trans-boundary watershed. The use of Generalized Linear Mixed Models (GLMM) with Beta distribution to predict the spatial index is also a robust statistical approach.
• Weaknesses for Standard Q1: The concept of integrating STI and NEI formulas using remote sensing and GIS data is already common in global hydrology literature (e.g., using UNESCO reference manuals or USLE/SDR-based empirical models).

***AUTHOR RESPONSE*** The novelty of this paper, as we have now re-emphasized, is the trans-boundary- nature of the habitat studied, the simultaneous considerable or multiple parameters influencing both catchment erosion and sediment transport processes; and the evaluation of the relative role of positive drivers vs mitigatory parameters, especially with incorporation of predictive modeling. Most previous studies examined just single parameters in isolation, and ven then just in one response direction.. I have also added the statement in the introduction thus: : “***The specific novelty is in simultaneously incorporating multiple LULC and geomorphic-hydrological parameters in the erosion equation with the deliberate aim of evaluating the relative effects of the positive drivers vs the mitigating factors, on the erision parameter itself, then using statistical modeling to compare across sub-watersheds to guide spatial prioritization for mitigation action.”***

The chosen analysis scale is the administrative level (district), rather than the natural biophysical scale (purely sub-watershed). This reduces its hydrological scientific value for Q1 journals because erosion and sedimentation processes do not follow political administrative boundaries.

***AUTHOR RESPONSE***: We agree with the reviewer that assessment and evaluation of phenomena such as erosion, sediment transport and deposition, though may be influenced by humans, largely occurs irrespective of consideration for human-delineated administrative boundaries such as counties.

Therefore all analyses has now been conducted at the sub-watershed scale, with the new geospatial analysis delineating a total of 10 rather than just the previous 6.

Any allusion to administrative units is now made only in as far as the sub-watersheds intersect or overlap the administrative units. This is because, ultimately, any conservation of rehabilitation action for redressing degradation caused by poor LULCs must necessarily be anchored within administrative demarcations

2. Synchronization Analysis (Problem, Objective, Method, Results, and Discussion)
Overall, the logical flow of this manuscript is quite coherent, but there are fatal inconsistencies (contradictions) between the hypotheses/objectives and the empirical results obtained:
A. Major Inconsistency
• Objectives/General Expectations vs. Actual Results: In the Introduction, the author states the general expectation/hypothesis (a) that districts covered by a larger proportion of the upstream slope of the watershed will contribute more to the scale of erosion and sedimentation.
• However, in the Results and Discussion section, it was found that the size of the upstream watershed (TUA) was insignificant for net erosion or sediment transport. In fact, the best model selected using the dredge AIC procedure actually discarded the upslope area (TUA) variable.

• Critique: The authors failed to revise the initial hypothesis narrative or were insufficiently thorough in analyzing why this traditional upstream-downstream hypothesis failed in the Lake Victoria watershed. The authors only briefly mention that the effect is offset by terrain ruggedness, but this weakens the theoretical foundation established in the introduction.

***AUTHOR RESPONSE***: The hypotheses have now been more streamlined and clearly stated in consistency with the study’s objectives. In particular, I have now clarified that I expected the watershed upslope size to be directly proportional with each of the three measured model response parameters – net erosion, sediment transport index and overall erosivity potential. In the results following the analyses, we observed that sub-watershed area was indeed directly proportional to sediment transport index (which is conventional) as well as total erosivity potential, but was not significantly determined by net erosion itself. I discuss this outcome in the discussion, including the possibility that in terms of erosion *perse*, the influence of upstream areas size might be mitigated by land cover elements such as forests and wetlands, whereas upslope area size may be more prominent in the case of STI because the scale of STI is determined more by geomorphic factors in spite of LULCs. In other words, large watersheds with sound anthropogenic land use practices may be less vulnerable to erosion than amsaller ones with near-bare ground with steeper slopes.

Just to add, as a side note, that analyses and tests are generally applied to data so as to test and subsequently either prove or disprove hypotheses. In this case, the hypothesis was disproved by the results of the experimental/analytical test procedure. This is normal in science.

B. Synchronization of Other Components
• Problem vs. Objective: Synchronized. The problem of Lake Victoria degradation due to sedimentation from upstream activities is clearly mapped to underpin the objectives of mitigation priority zoning.
• Method vs. Result: Synchronized. The use of the NEI, STI, and ErPot formulas is mathematically translated into a district ranking map (Figure 4) and GLMM regression graphs (Figures 5, 6, 7).

3. Critical Points and Technical Errors in the Manuscript
1. NEI Determination Method (Modified Formula): In the NEI equation:

The author sums variables with different units (area (km2), slope degree, elevation meters, and percentage). Although standardized logarithmic transformations are mentioned, this direct summation formula for different types of biophysical parameters is highly unusual and prone to index bias.

**AUTHOR RESPONSE:** The function has been rectified and is now presented as the ratio of the product of positive-driver parameters to that of the product of mitigator-parameters. In logarithmic/mathematical terms, it translates to the sum of logarithms of the positive-driver parameters minus the sum of logarithms of the mitigator-parameters.

2. Typographical/Textual Error in the Discussion Section:
In the final column (line 461), the text states: "Forest cover, high vegetation cover, high elevation, and agriculture on the slopes are further exacerbated by the very high population density, given that this is the smallest district in the Lake Victoria watershed...".
However, in the previous sentence, the author is discussing four districts (Kisumu, Kericho, etc.). The phrase "this is the smallest district" (singular) creates confusion as to which district is being referred to (according to real-world Kenyan data, Vihiga or Kisii are the smallest, not Kericho).

These have now been corrected

3.Limitations of Administrative Scale:
The authors acknowledge in the limitations section that this model is inefficient at the local/household scale. For PLOS ONE, the implications of ignoring plot-level management (terracing, hedgerows) should be emphasized as a limitation to the validity of this macro model.

erosion relationship paradigm.

**AUTHOR RESPONSE:** The section on study limitation is no longer part of the paper

4. Conclusion: Eligibility for PLOS ONE Journal
Recommendation: Major Revision.
This manuscript is ELIGIBLE for publication in PLOS ONE, but NOT in its current form.

This article has high public benefit value (according to PLOS ONE's inclusiveness criteria) because it produces a priority map of districts (Nyamira, Kisii, Kisumu, Kericho, and Busia) for practical conservation action.

5. Mandatory Improvements for Authors:
• Revision of the Hypothesis Paradigm: Authors should reword the final section of the introduction to avoid presenting a hypothesis that is completely rejected by their own statistical analysis, or explicitly state from the outset that this study challenges the traditional watershed-erosion relationship paradigm.

**AUTHOR RESPONSE:** The hypothesis has now been the redrafted to more clearly indicate that the expectation was to see sub-watershed upslope size being directly proportional to each of the individual three parameters assessed. This has now also been re-emphasized toward the terminal ends of Introduction. For instance, I have now added a statement in the Introduction thus: ***“Nonetheless, such sediment removal and transport may also be considerably cushioned by rougher terrain, or buffered by enhanced vegetation cover or wetland habitats which serve to either reduce sediment size, per capita amount, or movement speed^[33, 36, 37]^. This is more likely so if such buffering features constitute a proportion of the drainage basin, a scenario which may at times mitigate the conventional expectation of direct proportionality between watershed area size and discharge volume of water as a medium of surface erosion”.***

• Justification for the NEI Formula: Provide a strong scientific argument or literature citations as to why the distribution of the index by summing the logarithmic parameters is hydrologically valid.

**AUTHOR RESPONSE:** The function has been rectified and is now presented as the ratio of the product of positive-driver parameters to that of the product of mitigator-parameters. In logarithmic/mathematical terms, it translates to the sum of logarithms of the positive-driver parameters minus the sum of logarithms of the mitigator-parameters

• Visual Language Improvements: Ensure the maps in Figures 3 and 4 have a clearer resolution (especially the overlapping district name text).

**AUTHOR RESPONSE:** Figures 3 and 4 have now been reproduced after the new analyses, and now have higher resolution, color separation and legibility.

• Technical Clarification on Software Version: Correct the QGIS version on line 154 ("QGIS, v. 3/4/8" is not standard; use a specific version such as v. 3.28 or v. 3.34).

**AUTHOR RESPONSE:** This has now been corrected to v. 3.44.6

• Data Availability Statement: Provide a link or attachment of the tabular data for the 19 districts used as input for statistical modeling to meet PLOS ONE data transparency standards.

**AUTHOR RESPONSE:**. I have now provided a tabulated information in supporting material, detain the type, nature and source of the LULC datasets used in the analyses. This includes the websites for the sources of the metadata used in the analyses are now provided, for the institutions that do not have any raw-data sharing restrictions open public access restrictions

Xxxxxxxxxxxxxxxxxxxxxxxxxxxxxxxxxxxxxxxxxxxxxxxxxxxxxxxxxxxxxxxxxxxxxxxxxxxx

**Reviewer #2:**

Dear Author,

This manuscript, titled " Using linear modeling and remote sensing to identify priority spatial and parameter based mitigation measures against erosion and sediment movement within Lake Victoria watershed in Kenya” presents a highly interesting ideas for the management of the Lake Victoria watershed, but there are still some substantial weaknesses in almost all major parts of the manuscript.

Title
This manuscript's title is too long (26 words) and lacks interest. It should be no more than 20 words, convey the novelty immediately, and avoid excessive verb use. Please improve it.

It also uses uncommon terms, such as "sediment movement," which is commonly known as "sediment transport."

**AUTHOR RESPONSE:**. The title has now been shortened (22 words) sand more refocused. The term ‘sediment movement’ has now been replaced with ‘sediment transport’ throughout the paper

Abstract
An abstract generally consists of the research problem, the gap to be filled, the research objectives, the methodology, the main findings, and the implications. However, your abstract still needs improvement because it doesn't explicitly discuss all of these aspects, thus requiring revision to improve clarity and scientific focus. Check the manuscript writing guidelines in the Plos One Journal. Improvements include the following:
1. The abstract is very long (around 316 words) and explains the predictor variables in too much detail.

**AUTHOR RESPONSE**: The abstract has now been shortened to 300 words and all the structure and wording clarified to summarize the study, ensuring that all the key elements required in an abstract are presented succinctly.

2. The research gap is not clearly and explicitly stated.

**AUTHOR RESPONSE**: This has now been more clearly stated (second sentence)

3. The research objective is unclear, making it difficult for readers to understand the research objectives.

**AUTHOR RESPONSE**: This has now been more clearly stated (third sentence)

4. The novelty of the research is also unclear; it doesn't present what it presents.

**AUTHOR RESPONSE**: This has now been more clearly stated (final sentence). But this has ben more expounded in the introduction section. I have also added the statement in the introduction thus: : “***The specific novelty is in simultaneously incorporating multiple LULC and geomorphic-hydrological parameters in the erosion equation with the deliberate aim of evaluating the relative effects of the positive drivers vs the mitigating factors, on the erision parameter itself, then using statistical modeling to compare across sub-watersheds to guide spatial prioritization for mitigation action.”***

5. Line 15 - 21: This sentence is too long and too many focused. Please rewrite it and break it into several sentences. My suggestion is to revise and rewrite it to be more concise and focused.

**AUTHOR RESPONSE**: The sentence has now been considerably shortened for precision

Introduction
In the Introduction, the author discusses extensively: watershed degradation, Lake Victoria sedimentation, the importance of watershed conservation, and the impacts of land cover change. However, he fails to provide an adequate state-of-the-art overview of the methodology used.

**AUTHOR RESPONSE**: The methodology used here such as in using the formula to evaluate net erosion indicator and overall erosivity potential, is an innovative one and therefore not much literature exists to cite here. However the method for assessing STI is widely available and conventional and is dely cited in the corresponding section of the paper

1. This manuscript relies on GIS models, remote sensing, generalized linear modeling, and erosion-related indices. The Introduction should include a more comprehensive review of existing methods. However, several methods or approaches are used but not justified, such as Landsat Imaging, Generalized Linear Models (GLM), erosion, and sediment transport. How have previous studies analysed erosion and sedimentation? What models were used?, and why did you choose these models and the justification?

2. My suggestion is that the Introduction should include a paragraph or several discussing the state-of-the-art in erosion and sediment modeling. Then, a paragraph on statistical modeling approaches, including GLM.

Xxxxxxxxxxxxxxxxxxxxxxxxxxxxxxxxxxxxxxxxxxxxxxxxxxxxxxxxxxxxxxx

3. Why use counties as the unit of analysis instead of sub-watersheds? This should have been addressed in the Introduction. In fact, the erosion and sediment transport processes occur at the watershed/sub-watershed scale. It should be explained in the Introduction.

***AUTHOR RESPONSE***: All analyses has bnow been conducted at the sub-watershed scale, with the new geospatial analysis deilineating a total of 10 rather than just the previous 6.

Any allusion to administrative units is now made only in as far as the sub-watersheds intersect or overlap the administrative units. This is because, ultimately, any conservation of rehabilitation action for redressing degradation caused by poor LULCs must necessarily be anchored within administrative demarcations

4. The gap is still unclear, what hasn't been researched or known in previous research that will be filled in this study? Or what are the shortcomings of existing methods?

**AUTHOR RESPONSE:** The novelty of this paper, as we have now re-emphasized, is the trans-boundary- nature of the habitat studied, the simultaneous considerable or multiple parameters influencing both catchment erosion and sediment transport processes; and the evaluation of the relative role of positive drivers vs mitigatory parameters, especially with incorporation of predictive modeling. Most previous studies

Methodology
1. You have presented the dataset used in this research, but it is not systematically presented, making it difficult to understand. Information regarding data sources, acquisition period, spatial resolution, and analytical objectives is scattered throughout the Methods section. A table summarizing the following should be provided: data sources, spatial resolution or map scale, data period, data format, and data function in the analysis.

**AUTHOR RESPONSE:**. I have now provided a tabulated information in supporting material, detain the type, nature and source of the LULC datasets used in the analyses.

2. The methodological workflow is difficult to follow because it is presented in great detail and lacks a diagram of the overall analytical framework. The paper has a fairly complex workflow (DEM processing, LULC extraction, STI, NEI, ErPot development, GLM analysis, prioritization), but the reader does not see the relationship between these steps. A flow diagram should be added.

A flow diagram has now been provided

3. Was the LULC generated independently from Landsat? Or did you use LULC products available from other sources? This is unclear and needs to be improved.

**AUTHOR RESPONSE:** We have now clarified in te text that the LULC data were obtained from separate data sources to the landsat imagery data, which was exclusively online from EarthExplorer

4. If the LULC was generated from Landsat, it should explain: acquisition year, number of scenes, month, classification method used, validation method, and classification accuracy.

**AUTHOR RESPONSE:** LULC data were not acquired through Landsat, they were from various institutional sources for the various Kenyan counties overlapping/intersecting the delineated sub-watersheds. I have now provided a tabulated information in supporting material, detain the type, nature and source of the LULC datasets used in the analyses.

5. Was Landsat used only for specific variable extraction? If so, specify which variables.

**AUTHOR RESPONSE** Landsat was used as a source only for the satellite imagery scenes. This is now in the table provided in the supporting materials secction

Results
1. The results are too descriptive and do not emphasize the key findings.

**AUTHOR RESPONSE:** Without specification of any particular point or statement, subsection or paragraph by the reviewer, it is challenging to identify where action should be taken. However, extra care has been taken to present the results as they were obtained from the corresponding analysis procedures and steps

2. What is the **most dominant controlling factor**?: Is crop cover the largest driver of erosion? How much does it contribute compared to slope? These findings should be presented.

**AUTHOR RESPONSE**: Crop cover size was the most significant overall positive driver while forest and wetland cover were the most significant mitigating factors against erosion and sediment transport in general. We have now included this deductive idea in the results as well as discussion and conclusion sections

3. Figure 2 is more suitable as a supplementary source and should be replaced with a research flowchart.

**AUTHOR RESPONSE:** The former Figure 2 has now been considerably streamlined and summarized for precision, and is now presented a panel that is part of an overall schematic flow diagram which summarizes the geoprocessing and general analysis and modeling work in the study, just as was suggested by the reviewer

4. The legend in Figure 4 is illegible, with the font being too small. Please correct it for readability.

**AUTHOR RESPONSE:** The legend for Fig 4 has now been more streamlined to emphasize only key parameters, and is now in more readily legible font.

Discussion
1. The discussion is too descriptive.

**AUTHOR RESPONSE:** Without specification of any particular point or statement, subsection or paragraph by the reviewer, it is challenging to identify where action should be taken. However, extra care has been taken to strengthen points discussed by supporting them with evidence or results obtained

2. Lack of comparison with previous studies. Your key findings should be compared with previous research, whether they are in line with, or different from, and so on.

**AUTHOR RESPONSE:** This has been implemented

3. The subsection titled "Zonal prioritization and focus for catchment restoration and management" appears inconsistent between the results and the methods. The methods do not clearly explain how the prioritization procedure was carried out. How did you conduct this prioritization, and what criteria were used? Please complete the methodology.

**AUTHOR RESPONSE:** This has now been more clearly streamlined to reflect the new analyses which are now based on the delineated sub-watersheds as the spatial units rather than on administrative focal units as was done previously. We also include in the discussion that such prioritization was based on the comparative scales or magnitudes of net erosion indices (NEI), sediment transport indices (STI) and overall erosivity potential (ErPot). Prioritization is outlined based on each of the measures in turn/separately

Conclusions
The Conclusion section adequately summarizes the findings but does not sufficiently emphasize the broader scientific contributions of this study.

Authors are encouraged to strengthen the conclusions by highlighting how the proposed framework advances watershed prioritization and erosion management research **beyond the specific case study of Lake Victoria**. What is the paper's primary scientific contribution?

**AUTHOR RESPONSE:** The broader scientific contribution of the study has now more succinctly elaborated in the Conclusion section. We have added the following statement: ***“These results are readily applicable in a broader sense as a foundation for trend-monitoring initiatives on erosion and sediment transport processes, and controlling key LULC linkages towards staving off the rate of deposition across the Lake Victoria region as a whole, and in similar tropical lacustrine habitats worldwide.”***

Kindly Regards
Reviewer

6. PLOS authors have the option to publish the peer review history of their article ([what does this mean?](https://track.editorialmanager.com/CL0/https:%2F%2Fjournals.plos.org%2Fplosone%2Fs%2Feditorial-and-peer-review-process%23loc-peer-review-history/1/010f019f4ab8af71-9ecb38e6-cc7c-4780-9b97-d33426ff339c-000000/5N7UZ8XTE3GC-InlGeryrQ7ReA3qRYRIUPkFWG0FeKM=258)). If published, this will include your full peer review and any attached files.


If you choose “no”, your identity will remain anonymous but your review may still be made public.


**Do you want your identity to be public for this peer review?** For information about this choice, including consent withdrawal, please see our [Privacy Policy](https://track.editorialmanager.com/CL0/https:%2F%2Fwww.plos.org%2Fprivacy-policy/1/010f019f4ab8af71-9ecb38e6-cc7c-4780-9b97-d33426ff339c-000000/aZ8uQuyCZab0DdFperkW5Fumw7y61rEI3vzzlZhGq1c=258).

Reviewer #1: **Yes: Irmadi Nahib**

Reviewer #2: No
